# Predicting Natural Evolution in the RBD Region of the Spike Glycoprotein of SARS-CoV-2 by Machine Learning

**DOI:** 10.3390/v16030477

**Published:** 2024-03-20

**Authors:** Yiheng Liu, Zitong He, Liyiyang Jia, Yiwei Xue, Yuxuan Du, Huiwen Tan, Xianzhi Zhang, Yu Ji, Yigang Tong, Haijun Xu, Luo Liu

**Affiliations:** 1 College of Life Science and Technology, Beijing University of Chemical Technology, Beijing 100029, China; 2021201127@buct.edu.cn (Y.L.); tongyigang@buct.edu.cn (Y.T.); 2 College of International Education, Beijing University of Chemical Technology, Beijing 100029, China2021090013@mail.buct.edu.cn (H.T.); 3 College of Information Science and Technology, Beijing University of Chemical Technology, Beijing 100029, China; xianzhizhang723@gmail.com; 4 College of Mathematics and Physics, Beijing University of Chemical Technology, Beijing 100029, China

**Keywords:** SARS-CoV-2 RBD, machine learning, timestamping algorithm

## Abstract

Machine learning (ML) is a key focus in predicting protein mutations and aiding directed evolution. Research on potential virus variants is crucial for vaccine development. In this study, the machine learning software PyPEF was employed to conduct mutation analysis within the receptor-binding domain (RBD) of the Spike glycoprotein of SARS-CoV-2. Over 48,960,000 variants were predicted. Eight prospective variants that could surface in the future underwent modeling and molecular dynamics simulations. The study forecasts that the latest variant, ISOY2P5O1, may potentially emerge around 17 November 2023, with an approximate window of uncertainty of ±22 days. The ISOY8P5O2 variant displayed an increased binding capacity in the dry assay, with a total predicted binding energy of −110.306 kcal/mol. This represents an 8.25% enhancement in total binding energy compared to the original SARS-CoV-2 strain discovered in Wuhan (−101.892 kcal/mol). Reverse research confirmed the structural significance of mutation sites using ML models, particularly in the context of protein folding. The study validated regression methods (SVR, RF, and PLS) with different data structures. This study investigates the effectiveness of the “ML-Guided Design Correctly Predicts Combinatorial Effects Strategy” compared to the “ML-Guided Design Correctly Predicts Natural Evolution Prediction Strategy”. To enhance machine learning, we created a timestamping algorithm and two auxiliary programs using advanced techniques to rapidly process extensive data, surpassing batch sequencing capabilities. This study not only advances machine learning in guiding protein evolution but also holds potential for forecasting future viruses and vaccine development.

## 1. Introduction

**Figure 1:**
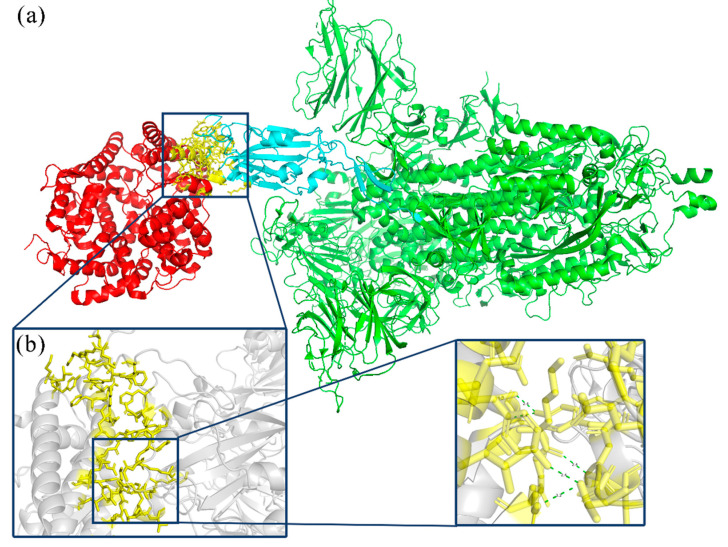
Interaction of SARS-CoV-2 Spike glycoprotein and ACE2 receptor. (**a**) RBD-ACE2 interaction model: the red part is ACE2; the blue part is the Spike glycoprotein RBD region; the green part is the Spike glycoprotein; and the yellow part is the RBD-ACE2 binding region. (**b**) RBD-ACE2 interaction interface.

Severe acute respiratory syndrome coronavirus 2 (SARS-CoV-2) is a coronavirus that has caused coronavirus disease (COVID-19) worldwide since mid-December 2019 [1]. As of 22 September 2023, according to data from the World Health Organization, SARS-CoV-2 has infected a total of 761,768,995 people globally, resulting in 6,784,168 fatalities. The ongoing COVID-19 pandemic is the most severe public health threat of this century. As of 24 September 2023, the China National Biological Information Center’s New Coronavirus Information Database (CNCB-NGDC) contained a total of 167,963 mutant strains. The Spike glycoprotein RBD region has a close relationship with the transmission mechanism of the virus and could significantly impact the infectivity and drug resistance of SARS-CoV-2. Consequently, it represents the primary target of interventions aimed at the SARS-CoV-2 antibody. Furthermore, the RBD region of the Spike glycoprotein of SARS-CoV-2 undergoes continuous mutations, which lead to a considerable decline in the activity of various monoclonal antibodies. Research by Elisabetta Cameroni et al. found that most monoclonal antibodies that are directed against the receptor-binding motif lost in vitro neutralizing activity against Omicron, with only 3 out of 29 monoclonal antibodies retaining unaltered potency [2]. Conversely, Shahai Ou et al. observed that neutralizing antibody potency in certain three-shot inactivated vaccines was lower than that of the D614G strain, which includes Omicron. The neutralizing antibody levels against the mutant strains decreased by around 4–6 times [3]. Therefore, it is valuable to conduct Natural Evolution mutations in the RBD region of SARS-CoV-2, and this prediction is of great significance to the development of related drugs and vaccines [4]. SARS-CoV-2 mediates viral entry into cells by binding the Spike glycoprotein carried on its surface to the ACE2 receptor on human cells. Figure 1 presents the interaction between the Spike glycoprotein and the ACE2 receptor. ACE2 is a homologous molecule of angiotensin-converting enzyme (ACE), a carboxypeptidase that degrades angiotensin II peptide on the cell membrane surface [5]. The receptor binding domain (RBD) of the new coronavirus spike protein is composed of 192 amino acid residues. The Spike glycoprotein predominantly binds to the peptidase domain (PD) of the host cell receptor ACE2 via RBD. There is a receptor binding sequence (RBM) on the RBD, which will specifically recognize the PD located in the extracellular domain of ACE2, but will not affect ACE2 function [5].

In recent years, machine learning (ML) technology has played an important supporting role in protein-directed evolution work, and thousands of proteins have been successfully designed using various software tools [6]. Directed evolution refers to simulating Natural Evolution in the laboratory, artificially generating a large number of mutants through random mutation and recombination, and conducting purposeful screening to obtain the required proteins [7]. Combining machine learning with directed evolution can reduce the cost of experimental testing of protein variants and obtain experimental results [8]. 

Through exploring various mutations, machine learning has the capability to effectively comprehend extensive sequence ranges to recognize enhanced proteins, which can then offer numerous solutions to engineering predicaments. In April 2019, Zachary Wu’s team affirmed this method in their investigation into the empirical fitness of human GB1-binding proteins, substantiating that machine learning-guided directed evolution has higher fitness than variants discovered by other directed evolution methods [6]. They developed an approach to employing a single enzyme for generating two enantiomeric products, which were anticipated by examining the database of functional enzymes [6]. To ascertain selective catalysis, they introduced seven mutations in two successive rounds of evolution. In November 2020, the research group of Sandipan Chakraborty from Amity University evaluated the binding efficiency of RBD variants with ACE2 through protein-protein docking and binding free energy calculations [9]. Studies have shown that structural changes in some RBD regions can lead to increased binding affinity for SARS-CoV2, which may be due to efficient receptor recognition mechanisms leading to high infection rates. Thus, investigating the structure of the Spike glycoprotein RBD region of mutant viruses around the world from large genome libraries and using machine learning methods to predict the direction of Spike glycoprotein mutations for early response plays an important role in controlling the spread of the virus [10]. Joseph. M.T. et al. used deep mutation learning to predict combinatorial mutations for ACE2 binding and antibody escape into the SARS-CoV-2 receptor binding domain. In this study, we developed DML, a machine learning-guided protein engineering approach, to determine the impact of combinatorial mutations in the SARS-CoV-2 RBD on ACE2 binding and antibody escape. In DML, machine learning models are trained on thousands of tagged RBD variants obtained from libraries. Screening makes highly accurate predictions in the sequence space of billions of RBD variants, which is larger than experimental screening alone. several orders of magnitude [11].

The PyPEF machine learning framework is a Python-based framework for protein engineering that utilizes machine learning techniques in conjunction with signal processing and statistical physics principles to carry out data-driven analysis [12]. PyPEF can be trained on small and medium-sized datasets. It can quickly complete more than 500,000 recombination mutation predictions on a personal microcomputer. It supports a variety of regression models, including support vector regression (SVR), random forest regression analysis (RF), and multi-layer perceptron (MLP). PyPEF has the capability to offer formidable solutions for sequence exploration and combination hurdles encountered in protein engineering by means of comprehensive computer screening of sequence space [12]. Using time as a variable for machine learning, it is valuable and feasible to correlate time with specific physical and chemical properties to forecast Natural Evolution mutations of SARS-CoV-2. This research obtained both wild-type and mutant sequences of the Spike glycoprotein of SARS-CoV-2 from the NCBI database. Moreover, 446 RBD region sequences were subsequently attained after screening and interception. The mutant strains were compared to the standard strain using the “SeqExplorer” program. The mutation code for each mutant strain was obtained, and a timestamp was established to quantify the earliest time of the variant. Finally, a time and mutation residue dataset was generated to build a training set for a machine learning model. Three regression methods, PLS, RF, and SVR, were used to train the model. Based on R^2^, Spearman coefficient, and training time, an optimal model was selected to further summarize the evaluation method based on previous research. Iterative mutagenesis was also employed to predict future recombination variants. The prediction results were used to select some possible models for further modeling. The target variant is validated using molecular dynamics simulations (MD) to determine its binding ability. The study initially explores the strategy of utilizing machine learning to forecast Natural Evolution mutation trees.

## 2. Materials and Methods

### 2.1. Dataset Collection

The data source for this study is the website of the National Center for Information Biology (NCBI, https://www.ncbi.nlm.nih.gov/; last accessed: 16 December 2023). Obtain the complete Spike glycoprotein sequences of “mutants of concern” (VOI) and “new mutant strains under monitoring” (VUM) from September to December 2022, as well as all mutant strains from January to July 2023, for a total of 959 sequences. SeqExplorer was used to sort the sequences. Obtain the amino acid sequence of the RBD region of the new coronavirus, that is, amino acids 319 to 541 of the Spike glycoprotein of the new coronavirus, and manually screen to remove sequences containing a large number of continuous unknown amino acids.

### 2.2. Machine Learning

The following operations are mainly performed during the machine learning process:

#### 2.2.1. Data Format Organization

Adjust the data format used for machine learning to the format required by PyPEF software, store mutation data in CSV format, all data are arranged in the “mutation code-time” format, and the characters corresponding to variant discovery and sequencing time are The string is converted into a numerical value that can be operated on [12].

#### 2.2.2. Machine Learning Model Training

106 mutant strains and wild-type viruses were screened to build a model training set for machine learning. To develop models for adaptive prediction of sequence variation, identifying and generating appropriate features is crucial. These features, typically numerical, are transformed into corresponding fitness values through a mapping function. Subsequently, the generated sequence of numbers undergoes normalization before conducting the Fast Fourier Transform (FFT). Hence, the inputs for model training are represented as FFT sequence vectors. PLS, RF, and SVR were used as regression methods [12].

Among them, the model uses AAindex encoding. AAindex is a database used to describe the properties of amino acids. This database collects a large number of numerical indicators describing the biochemical and physicochemical properties of amino acids. These metrics can describe various properties of amino acids, such as solubility, hydrophobicity, charge, size, etc. Data in AAindex can be used in the fields of bioinformatics and computational biology to help researchers understand the relationship between protein sequence and structure and their connection to biological function [13].

The evaluation method is defined as follows by the PyPEF software designer [12].

(1) R^2^ coefficient

R^2^ is calculated by comparing the difference between the model-fitted data points and the real data points to measure the fit of the model. The value range of R^2^ is usually between zero and one. The closer to one, the better the model fits; that is, the more the independent variables explain the changes in the dependent variable.

(2) Spearman’s rank correlation coefficient

It is a statistic used to measure the monotonic relationship between two variables. The Spearman coefficient does not require a linear relationship between variables but is calculated based on the rank of the variables.

(3) Pearson correlation coefficient

It is a statistical indicator that measures the degree of linear correlation between two variables. It measures the strength and direction of the linear relationship between two variables. 

#### 2.2.3. Model Selection

After completing the training, PyPEF will display the model-related scores and select R^2^ and the Spearman coefficient as the criteria for determining the degree of model fitting in the initial screening stage. The two models obtained through preliminary screening were analyzed using the coefficient of determination R^2^ and the Spearman coefficient.

All existing variants were tried for prediction to confirm the fit more intuitively. After comparing the predictions with the actual situation, perform re-screening to obtain an optimal model.

#### 2.2.4. Prediction

Summarize the single-point mutations that appear in all variants, and use the command “PyPEF mkps” to rearrange the mutations. The final model of this program is utilized to identify substitutions at predetermined key positions through systematic scanning of the sequence landscape, thereby facilitating the computational exploration of recombinant variants. Given the infeasibility of exhaustively traversing the entire recombination space, particularly for highly substituted variants, the program employs a statistical Markov Chain Monte Carlo (MCMC) method. Leveraging the Metropolis-Hastings acceptance criterion, it simulates directional evolution to effectively identify favorable substitutions.

Iterations were performed using SnapGene, retaining the temporally most distant mutations. At the same time, the point mutations P12L, A19L, S46F, S48P, S50F, T51A, D80N, and K92N that appeared repeatedly in the original dataset were also retained in order to be more consistent with the characteristics of Natural Evolution. The variant frequency statistics are shown in Table S2.

### 2.3. Model Construction and Energy Calculation

Based on the prediction results, Snapgene software was used to edit the RBD region of the wild-type sequence, and the amino acids at the predicted mutation sites were replaced with the mutated amino acids. Import the new sequence into AlphaFold2 for modeling, change the name, adjust parameters, set the number of model cycles to 24 (num_recycles = 24), the number of models to 1 (num_models = 1; model_order = [1]), optimize the number of protein structures to 5 (num_relax = 5), and set the reference database to PDB100 (template_mode = PDB100) [14].

PyDock (https://life.bsc.es/pid/pydock/; last accessed: 16 December 2023) was used for molecular docking. The docking method is rigid docking, and the selected docking sites are shown in Figure S2 [15]. The program uses Fourier correlation theory to score potential complexes based on shape complementarity and favorable electrostatic interactions. PyDock provides a total binding energy defined by the sum of the electrostatic energy component and the desolvation energy component during the binding process. The online docking platform will sort according to the total binding energy to obtain the corresponding protein model [15]. 

## 3. Results and Discussion

### 3.1. Data Analysis and Discussion of SARS-CoV-2

The data necessary for this research were procured from the NCBI database (https://www.ncbi.nlm.nih.gov/; last accessed: 16 December 2023). The entire sequence of the Spike glycoprotein of the new coronavirus mutant strain was downloaded from the NCBI database. To guarantee efficient and accurate machine learning procedures in the next steps, data cleansing is mandatory. The primary reasons are twofold, as follows: Firstly, the correlated sequence is excessively protracted, which hinders ML’s precise analysis of the RBD locality. Secondly, the correlated sequence encompasses an abundance of inexplicable sequences. In order to facilitate data cleaning, SeqExplorer, a program for batch processing of data and files, was developed. It contains five functions, mainly designed for the data format required by PyPEF. Its functions are as follows:

1. Install the necessary Python extension plug-ins.
2. Intercept the initial and terminal amino acid residues to obtain the protein sequence.
3. Sequence comparison: Create a chart to compare sequence differences.
4. Date conversion: Convert string dates into numerical values.
5. Mutation writing: The mutation code will be inserted into the ‘*.fasta’ sequence file.

Amino acids 319–541 from the Spike glycoprotein were separately identified. The analysis revealed that the initial seven amino acids of the intercepted RBD region sequence set (amino acids 319–326 of the Spike glycoprotein) predominantly consist of consecutive unknown amino acids. In order to avoid the impact of unknown sequences on machine learning, these seven amino acids are not involved in subsequent predictions. After model simulation, it was found that this region is highly flexible, has a disordered structure, and is not involved in binding to the ACE2 protein, so this study will no longer recombine this part. The final sequence used for machine learning was amino acids 326 to 541 of the Spike glycoprotein. After removing repetitive sequences and retaining the earliest sequenced variant of this type of variant, a total of 106 amino acid sequences were obtained. 

**Figure 2:**
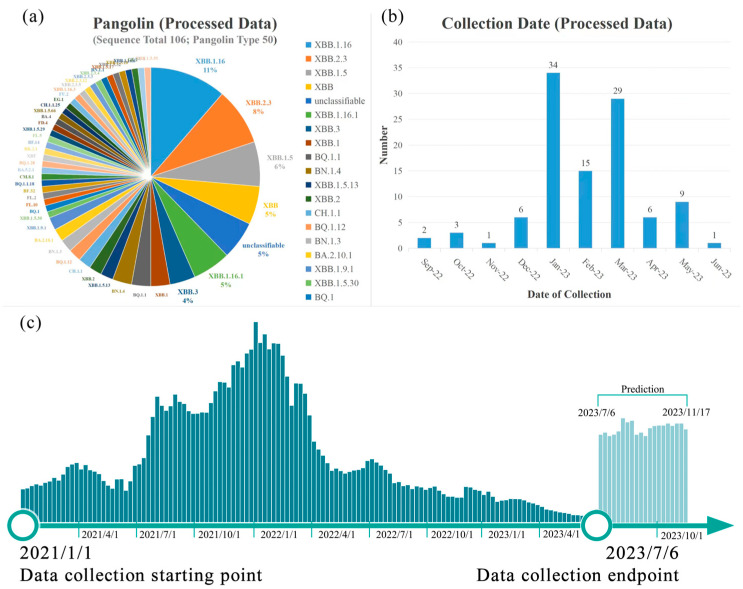
(**a**) Time and type distribution of mutant strains after screening. (**b**) Collection Date. This figure shows the distribution of the collected variants from September 2022 to June 2023. (**c**) Number of new coronavirus Spike glycoprotein sequences added every week from January 2021 to June 2023. The gray part is the predicted variant.

As shown in Figure 2a, the dataset comprises 49 distinct subtypes, with XBB.1.16 subtype (11%), XBB.2.3 subtype (8%), and XBB.1.5 subtype (7%) constituting the largest proportions [16]. XBB.2.3 is a newly emerging strain known as the “Omicron” mutant. According to the Chinese Centre for Disease Control and Prevention, XBB.2.3 was initially detected in China between 17 and 23 March 2023.

The 106 sequences that were screened yielded 123 mutations detected at 77 sites. Among them, multiple high-frequency repetitive mutations, such as D80N and K92N, appeared 104 times in 106 sets of data. The distribution of these variants over time is shown in Figure 2b.

It is noteworthy that during data collection, a considerably lower number of new Spike glycoprotein sequences of COVID-19 in 2023 was found compared to those of 2021 and 2022. Figure 2c showcases the weekly frequency of new Spike glycoprotein variants of COVID-19 from January 2021 to June 2023. It can be inferred that, due to decreased COVID-19 research, certain variants with low pathogenicity have not been documented.

### 3.2. Discussion on Machine Learning

The mutation prediction process primarily employs the PyPEF machine learning software (GitHub—Protein-Engineering Framework/PyPEF: PyPEF—Pythonic Protein Engineering Framework) [12]. Three regression methods—PLS, SVR, and RF—were analyzed. And the research on iterative evolution was optimized.

#### 3.2.1. Design of Virus Variant Emergence Timestamp

This study aims to utilize the emergence time of SARS-CoV-2 to fit certain physical and chemical properties. In this machine learning process, it is necessary to directly show the relationship between variant recombination and the time when it may appear. The mutation sequence is used as the input feature, and the time is used as the label. This is in line with the PyPEF program’s benchmarks [12]. However, it is noteworthy that time cannot be computed as a number in computer language but as a string. Despite its ability to gauge the variability of SARS-CoV-2 as an axis, time cannot be directly involved in the computation. Hence, a computable timestamp concept must be introduced. The initial timestamp is set as “1” on 1 January 2019, and it increases by “1” every day thereafter. Divide this number by seven to obtain a value expressed in weeks. Include decimals to retain pertinent date information. To prevent an overly long timestamp, divide the number by 10 to obtain a machine-learning-friendly value. Refer to the diagram below for the formula.

$$Dat=\frac{\sum_{i=1}^{n}i}{7\times 10}\;i:\;\mathrm{number}\;\mathrm{of}\;\mathrm{days}$$

In this study, time is quantified as a non-negative real number within 30. The function for timestamp conversion has been omitted and instead incorporated into the “date conversion” feature in the SeqExplorer program mentioned previously.

#### 3.2.2. Comparison of Three Fitting Methods

This study mainly uses the AAindex model of PyPEF for coding, uses a variety of different regression methods for calculation, and compares the three regression methods. Table 1 displays the fitting conditions of the different regression methods. Our findings suggest that the optimal models differ across the three regression methods, signifying their varying adaptability to the data.

Based on the coefficient of determination (R^2^)’s general classification, a moderate correlation is indicated by values ranging from 0.3 to 0.6, while a strong correlation ranges between 0.6 and 0.8 [17,18]. Upon analyzing the Spearman coefficient’s features, it can be inferred that all models display uniform monotonicity. However, the determination coefficient R^2^ indicates that none of these models accurately predict linear regression [18]. Thus, it has been established that this dataset exhibits monotonicity after partial AAindex encoding, with discernible patterns in nonlinear space. When processed on the same computer, SVR and PLS exhibit relatively short processing times, whereas RF regression fitting requires longer. Literature suggests that SVR outperforms PLS when processing high-dimensional and nonlinear data, while RP excels at handling large amounts of data [17]. Following these experiments, this study has determined the appropriate options for selecting data types, computational quantities, and regression methods. The combined effects of different data types and regression methods are displayed in Table 2. Following a comparative analysis, two models with better results in SVR regression have been selected for subsequent operations.

#### 3.2.3. Comparison between Model Prediction and Actual Situation

**Figure 3:**
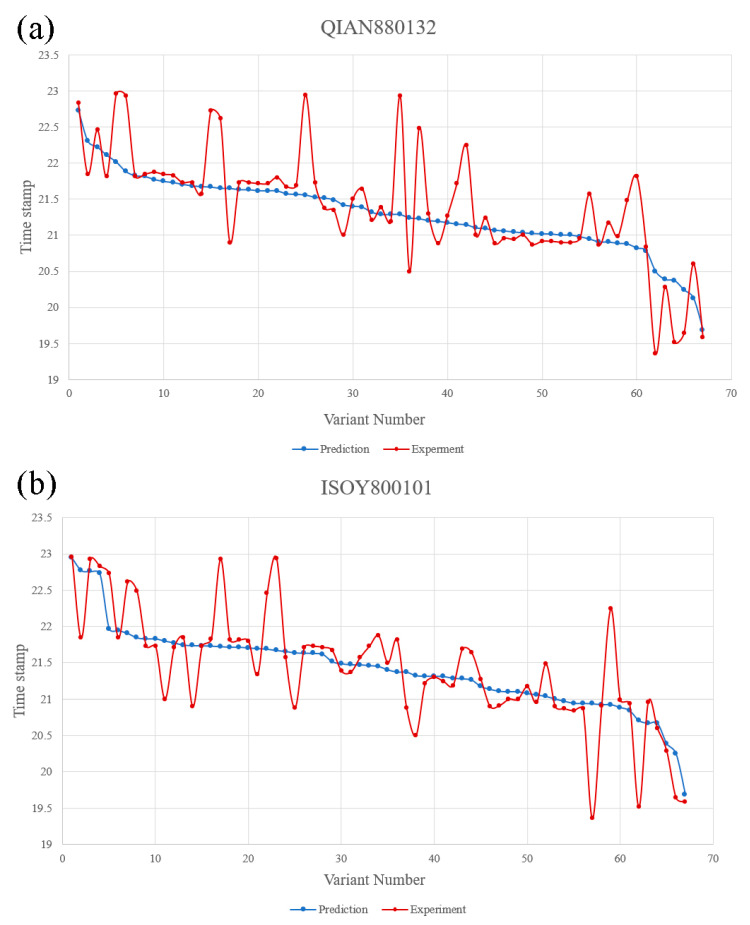
(**a**) QIAN880132 prediction situation: the light gray line is the prediction result; the blue gray line is the actual situation. (**b**) ISOY800101 prediction: the blue-gray line is the prediction result; the dark blue line is the actual situation. Detailed variant data can be found in Table S3.

QIAN880132 and ISOY800101 were used in SVR fitting mode to predict the appearance time of 106 variants in the dataset. The model regression chart is shown in Figure S1. The prediction results and the actual appearance time of the variants are shown in Figure 3.

Based on these predictions, it can be affirmed that the QIAN880132 model has an average deviation of 24 days, whereas the ISOY800101 model has an average deviation of 22 days. The QIAN880132 model is generally employed for predicting secondary structures, while the ISOY800101 model is used for detecting multiple bending features. The two models share certain similarities in their mechanisms, indicating that multiple variants could significantly impact the structure of the RBD region. Therefore, future forecasts will prioritize amino acids with noticeable alterations in the arrangement of the RBD area. Therefore, future forecasts will prioritize amino acids with noticeable alterations in the arrangement of the RBD area. Based on the average deviation, future predictions will be made using ISOY800101.

#### 3.2.4. Iterative Recombination Prediction of Viruses

This study tested various site combinations and conducted multiple rounds of prediction.

The initial set of predictions was based on the wild-type, and subsequent research revealed that variants P12L and A19S will emerge at a later time. By holding onto these two positions, there is potential for us to make new developments over an extended time frame.

The second set of predictions is based on including new variants of P12L and A19S as the original samples, based on the wild type. The deadline for predicting this set of samples has been extended to November 15th.

The original samples used in the third set have been modified. In addition to P12L and A19S, six loci that are commonly observed in the wild type have been incorporated into the original sample (S46F/S48P/S50F/T51A/D80N/K92N), each with a frequency of over 98%. 

Ultimately, more than 48,960,000 variants were predicted. A comprehensive analysis of some of these variants will be provided in subsequent sections.

Part of the results of the three sets of predictions are shown in Table 3.

The analysis of the findings indicates that there is a high frequency of P12L and A19S mutations, which may be attributable to alterations in structure and folding. Nevertheless, examination of the interaction model between the RBD region of the Spike glycoprotein of COVID-19 and the ACE2 receptor establishes that this factor exerts very little influence on binding. Based on the action of ISOY800101, it can be inferred that the presence of P12L and A19S sites may affect the stability of the free structure of the RBD region, potentially leading to the formation of new folds. Additionally, the farthest variant is forecast to occur by approximately 17 November 2023, suggesting its appearance within a range of +/−22 days after that date. At present, it is found that multiple similar mutations have occurred. For example, the 8P9Y and 8P99 series discovered in September 2023 are similar to the forecasted time and mutation situation. At the same time, the 7VYR_C and 7K9Z_E variants with high similarity were also reported on 29 November 2023 and 18 October 2023. (https://www.ncbi.nlm.nih.gov/protein/8P9Y_B; last accessed: 12 March 2024).

#### 3.2.5. Molecular Docking and Binding Energy Calculation

**Figure 4:**
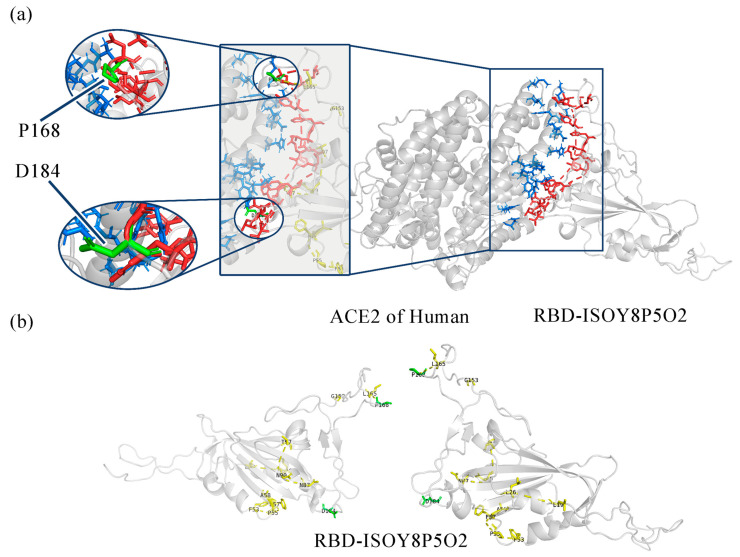
(**a**) ISOY8P5O2 combined model (**b**) ISOY8P5O2 single model.

From the prediction results, the eight variants with the farthest expected appearance time are selected. The model of the variant code ISOY0P5O2 is shown in Figure 4.

The total binding energy table (Table 4) was generated for the eight modeled variants using distance and angle constraints. Table 4 indicates that the wild type’s predicted total binding energy is −101.892 kal/mol, while the binding abilities of ISOY0P5O2 and ISOY8P5O2 have increased. The mutation code used was ISOY0P5O1. Based on the comparison of mutation sites in previous statistics, it is evident that the binding site region present in ISOY8P5O2 has the highest number of mutations and is in proximity to the recently uncovered coronavirus variants.

#### 3.2.6. The Potential Impact of Mutations

This study examined several locations of ISOY8P5O2 in Chakraborty. S’s research indicates that evolutionary analysis reveals five RBD variants, i.e., A30T, V49F, G149S, V165A, and S176P, are under strong positive selection bias [9,10].

Current research has found that A30T exhibits a lower affinity for ACE2. V165 is situated within the 482–485 loop and is associated with ACE2 recognition [9]. The research conducted has established that V165A functions to lower the binding capacity of the RBD region to the ACE2 protein. This outcome is due to alanine’s lack of ability to elevate the stability of β-Sheet, while leucine exhibits greater stability than alanine in β-Sheet and, thus, proves to be more effective in this context. These findings align with the research of Chakraborty, S. However, in light of Li Q. et al.’s research, it is possible for V165A (previously known as V483A) to generate resistance against mAb X593 and P2B-2F6. As a result of human interaction, V483A might become more prevalent in future SARS-CoV-2 strains [19,20,21] (source: https://nmdc.cn/nCov/structure?pdb=6LZG/; last accessed: 16 December 2023). 

A large number of high-frequency mutation sites (P19L/A26L/S53F/S55P/S57F/T58A/D87N/K99N) were selected for iterative prediction in this study. Among these, the Omicron and Deltacron mutations predominantly affect the S53F/S55P/S57F/T58A/D87N/K99N sites. Currently, analysis suggests that these mutations enhance the virus strains’ immune escape ability (source: https://www.bv-brc.org/view/VariantLineage/; last accessed: 16 December 2023) [22]. 

There are three mutations in ISOY8P5O2 related to the binding site, which are K99N, F168P, and G184D [23,24]. The earlier explanation for K99N has been covered. According to [25], F168P enhances the RBD region’s expression ability by lowering hydrophobicity. Additionally, G184D directly enhances the affinity between spike proteins and ACE2 [26]. However, ACE2 binding has strongly constrained G184, and this constraint may only be lifted if glycine transforms into aspartic acid.

The aforementioned mutations’ mechanism is moderately linked to the displayed binding energy. Upon combination, it may lead to an improvement in the stability and binding ability of SARS-CoV-2. These variants may appear in the future.

#### 3.2.7. Regression Model and Time Relationship

Based on the properties of the ISOY800101 model, the relationship between the model and the emergence time of new recombinant SARS-CoV-2 variants was analyzed. The property directly related to this model is the stability of different secondary structures. The RBD region contains multiple β-sheets, and PyPEF was fitted to the β-sheets. The study concludes that there is a positive correlation between the stability of the β-sheet and the generation of SARS-CoV-2 strains. The model is based on changes in β-sheets to predict the emergence of recombinant variants. Many of the mutations in this study were neutral mutations. Neutral mutations can set the stage for adaptation by providing a varied set of evolutionary starting points [27]. Additionally, this research identified that these mutations may have led to improved β-sheet stability. The naturally selected variants are presented in chronological order. Therefore, it is possible that the phenomenon attributed to emergence time and β-sheets is a result of epistatic coupling caused by natural selection.

Simultaneously, recombination prediction resembles a stochastic process, which can be exploited for certain predictions. This research implemented the stability of β-sheets as a quantitative measurement associated with time. The emergence time of certain sites corresponds with the augmented stability of β-sheets. These locations form an independent distribution of β-sheet stability. The distributions are combined to create a stochastic process akin to β-sheet stability [28]. The time dependence of the β-sheet results from the model’s nature and fitting circumstances. Consequently, the process follows a similar random distribution over time and is impacted by β-sheet stability. While it cannot be summarized by a unified mathematical model, there is some monotonicity evidenced in the fitting.

### 3.3. Strategy Optimization

This study adopted “the ML-Guided Design Correctly Predicts Combinatorial Effects Strategy” [29]. This strategy has been developed in preliminary studies to provide a framework that has some potential for practical application and is used for the directed evolution of proteins. However, the regression method and verification stage of predictions are insufficient for machine learning to simulate natural reorganization iterations. This study improves upon the framework by incorporating extra steps. Three primary steps have been added:

1. Iterative prediction

Iterative recombination prediction by machine learning can be closer to the characteristics of directed evolution by forming recombination mutations with more than five recombination points, which is conducive to improving the optimization of multiple properties at nonlinear sites. Thus, an iterative recombination evolution strategy based on multiple traits can be developed.

2. Variant site statistics

This process proves advantageous in analyzing complex iterative recombination and is useful for combining artificially complex sites. Its application partially confirms the suitability of the mechanism-based model for each site. Furthermore, it enables the production of related derivative studies on the amino acid mutation distribution and multi-point random process studies based on these factors.

3. Model analysis

The analysis of amino acid index is conducive to exploring the mechanism of the impact of combinatorial mutations. The analysis of the model is helpful to guide the mechanism analysis and the selection of reasonable verification methods. For example, if the model is related to structure, verification methods such as binding energy and folding free energy can be used. And if it is related to hydrophobicity, hydrophobicity prediction can be used to find relevant rules. The analysis results may provide new ideas for protein optimization and further use the properties of amino acids to evaluate the possible effects of certain mutations.

**Figure 5:**
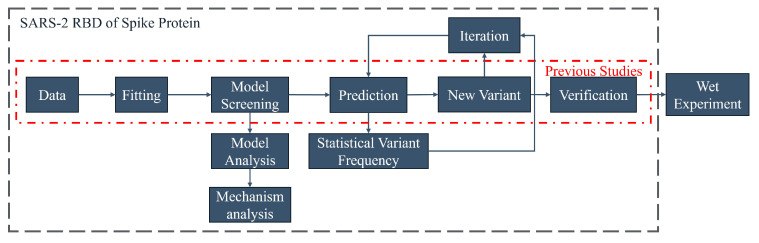
The picture shows the optimized “ML-Guided Design Correctly Predicts Combinatorial Effects Strategy”. Red dashed line: strategy before optimization. Blue dashed line: optimized strategy.

With the inclusion of three additional steps, this approach expands the prediction of Natural Evolution. The relevant model is first fitted by this program, using time as a parameter. Next, the program explores the Natural Evolution mechanism and predicts potential recombination variants in the future. Iterations are carried out to complete the relevant work with minimal calculation. The program has potential value for the early development of vaccines and monoclonal antibodies. The overall strategy is shown in Figure 5. 

## 4. Conclusions

SARS-CoV-2 is a highly pathogenic virus that spreads rapidly and poses challenges to the development of effective long-term drugs and vaccines due to its rapid mutation rate. To effectively combat SARS-CoV-2, predicting potential variants and conducting research, development, and technical reserves of drugs and vaccines in advance are crucial. In this study, a simulated Natural Evolution approach was devised based on “the ML-Guided Design Correctly Predicts Combinatorial Effects Strategy”. The strategy can aid in forecasting future virus variations and producing relevant vaccines. Furthermore, it has broadened the capabilities of the initial strategy. During the research objectives’ exploration, time was used as a parameter, and corresponding timestamps and conversion procedures were formulated. Simultaneously, this investigation assesses the distinctions between various regression techniques in PyPEF, which consist of support vector regression (SVR), random forest regression analysis (RF), and partial least squares regression (PLS). Subsequently, in combination with the network data collection, this research performed a prediction experiment on the naturally occurring recombination mutation of the RBD region of the SARS-CoV-2 spike protein, followed by the application of iterative prediction techniques for the first time. Screen the mutation results for eight variants that are expected in the future. Molecular dynamics simulations reveal that the variant, ISOY8P5O2, has an inclination to increase its binding capacity compared to the wild-type virus. It is anticipated that the ISOY2P5O1 variant will emerge between 17 and 22 November 2023. Although research forecasts regarding time are currently limited, as strategy optimization and the number of iterations increase, pertinent research will undoubtedly assist and guide the development of vaccines and new drugs.

## Figures and Tables

**Table 1:** Machine learning-fitting situation.

**(a)**					
**Index**	**R^2^**	**RMSE**	**Pearson**	**Spearman**	**Definition**
QIAN880132	0.36	0.60	0.69	0.61	Weights for the coil at the window position of −1
ISOY800101	0.42	0.57	0.67	0.59	Normalized relative frequency of the alpha-helix
KARS160113	0.26	0.65	0.54	0.53	Weighted domination number using the atomic number
GEIM800101	0.14	0.70	0.50	0.52	Alpha-helix index
ROSM880101	0.30	0.63	0.58	0.52	Side chain hydropathy, uncorrected for solvation
**(b)**					
**Index**	**R^2^**	**RMSE**	**Pearson**	**Spearman**	**Definition**
WERD780103	0.12	0.71	0.46	0.64	Free energy change of alpha(Ri) to alpha(Rh)
NAGK730103	0.15	0.69	0.42	0.61	Normalized frequency of coil
GEOR030101	0.35	0.61	0.61	0.59	Linker propensity from all datasets
AURR980102	0.29	0.64	0.55	0.57	Normalized positional residue frequency at helix termini N”
MIYS990105	0.14	0.70	0.43	0.57	Optimized relative partition energies—method D
**(c)**					
**Index**	**R^2^**	**RMSE**	**Pearson**	**Spearman**	**Definition**
CHOP780201	0.27	0.64	0.60	0.62	Normalized frequency of the alpha-helix
RACS820104	0.24	0.66	0.56	0.58	Average relative fractional occurrence in EL(i)
PONP800106	0.29	0.63	0.60	0.59	Surrounding hydrophobicity in turn
NAGK730103	0.17	0.69	0.48	0.56	Normalized frequency of coil
FUKS010111	0.33	0.62	0.60	0.55	Entire chain composition of amino acids in extracellular proteins of mesophiles (percent)

(a) SVR fitting results; (b) PLS fitting results; and (c) RF fitting results. The research is based on R^2^, the Spearman coefficient, and the Pearson coefficient to confirm its linear correlation and monotonicity. In machine learning prediction, usually, 0.33–0.67 is a medium correlation, and 0.33–0.67 is a strong correlation. Values of RMSE between 0.1 and 1 are acceptable [12]. Please visit (https://www.genome.jp/aaindex/, accessed on 19 March 2024) for the definition and meaning of AAindex [13].

**Table 2:** Combinations of different data types and regression methods.

	Time	Linearity	Data Volume	Data Continuity
PLS	−	+	−	+
SVR	−	−	+	−
RP	+	−	+	−

**Table 3:** Prediction results. Three rounds of iterative predictions were conducted based on the ISOY800101 model. A number of mutations considered meaningful were retained before each prediction. The main basis for determination is statistics at a relatively distant time. Finally, seven variants were intercepted.

Model	ISOY800101					
Iteration	-		P12L/A19S		P12L/A19L/S46F/ S48P/S50F/T51A/ D80N/K92N	
Classification	Variant	Day	Variant	Day	Variant	Day
1	P12L/A19S/ R83N/V158L/ F161P	2023/10/15	A23S/R83N/ V158L/F161P/ G177D	2023/11/17	R83N/V120P/ V158L/F161P/ G177D	2023/10/10
2	P12L/A19S/ R83N/V158L/ G177D	2023/10/14	A23T/R83N/ V158L/F161P/ G177D	2023/11/15	R83N/E146G/ V158L/F161P/ G177D	2023/10/8
3	-	-	R83N/E146G/ V158L/F161P/ G177D	2023/11/14	-	-

**Table 4:** Total binding energy table.

Variant	TOTAL_eneRST (kal/mol)	TOTAL_ene (kal/mol)
WT	−101.892	−44.99
ISOY0P5O1	−104.863	−36.789
ISOY0P5O2	−103.825	−40.992
ISOY2P5O1	−100.426	−50.709
ISOY2P5O2	−84.269	−41.02
ISOY2P5O3	−88.887	−41.348
ISOY8P5O1	−89.04	−40.848
ISOY8P5O2	−110.306	−39.196

## Data Availability

All the relevant data used in this study have been provided in the form of figures and tables in the published article, and all data provided in the present manuscript are available to whom they may concern.

## Supplementary Materials

The following supporting information can be downloaded at: https://www.mdpi.com/article/10.3390/v16030477/s1. Figure S1: Regression status of each model; Figure S2: Site selection for rigid docking; Table S1: Time-variation code table; Table S2: High-frequency mutation sites. Table S3: Validation Data.
