# Supplementary material for: Predicting Natural Evolution in the RBD Region of the Spike Glycoprotein of SARS-CoV-2 by Machine Learning
Source: Viruses. 2024 Mar 20;16(3):477. doi: 10.3390/v16030477 (PMC10974066; doi:10.3390/v16030477)
Supplement: Supplementary file 1 [file viruses-16-00477-s001.zip › viruses-2888739-supplementary.pdf]

Supporting Information

Forecasting Natural Evolution in the RBD Region of the Spike glycoprotein of SARS-CoV-2 by Machine Learning

Yiheng Liu, Zitong He, Liyiyang Jia, Yiwei Xue, Yuxuan Du, Huiwen Tan, Xianzhi Zhang, Yu Ji1, Haijun Xu* Luo Liu*

**Figure S1**	**Regression status of each model**	**P1**
**Figure S2**	**Site selection for rigid docking**	**P3**
**Table S1**	**Data set**	**P4**
**Table S2**	**High frequency mutation sites**	**P7**
**Table S3**	**Validation Data**	**P8**

ISOY800101

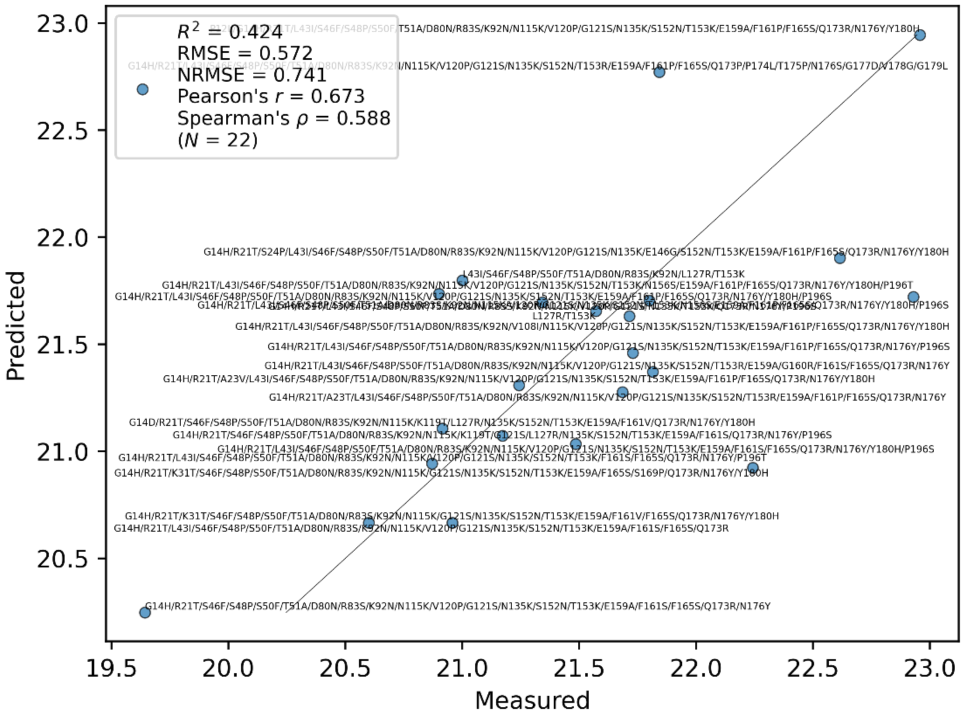

AURR980108

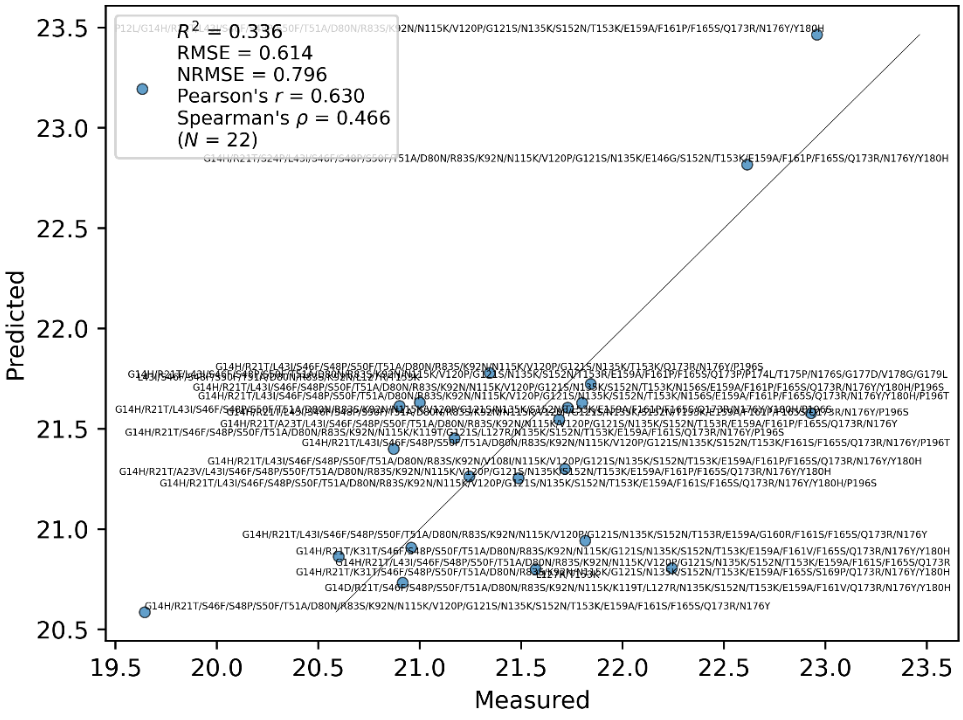

QIAN880132

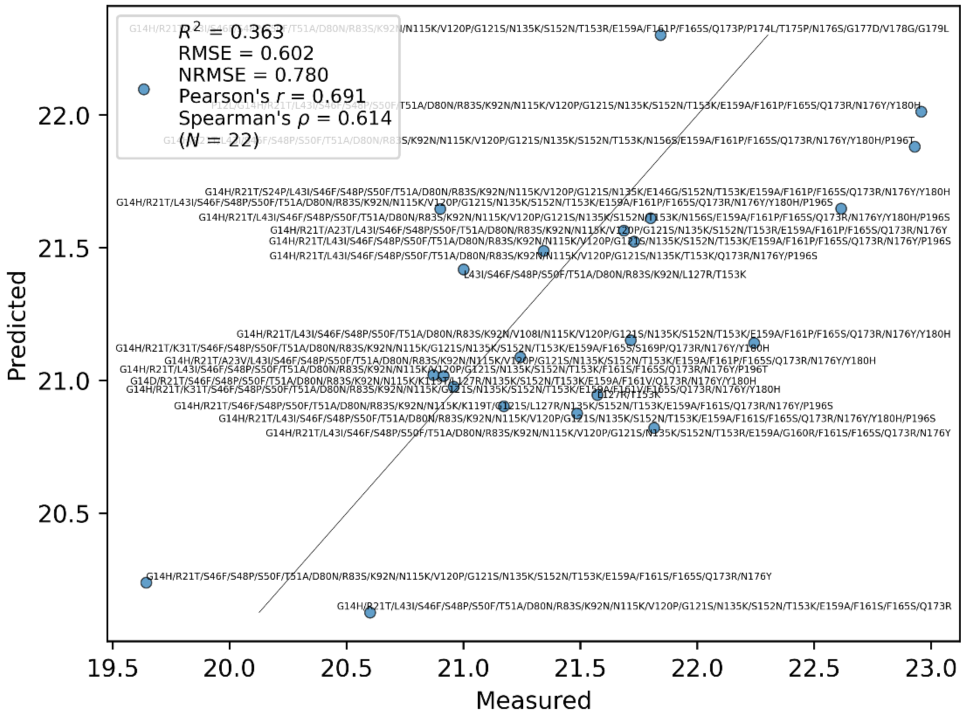

ROSM880101

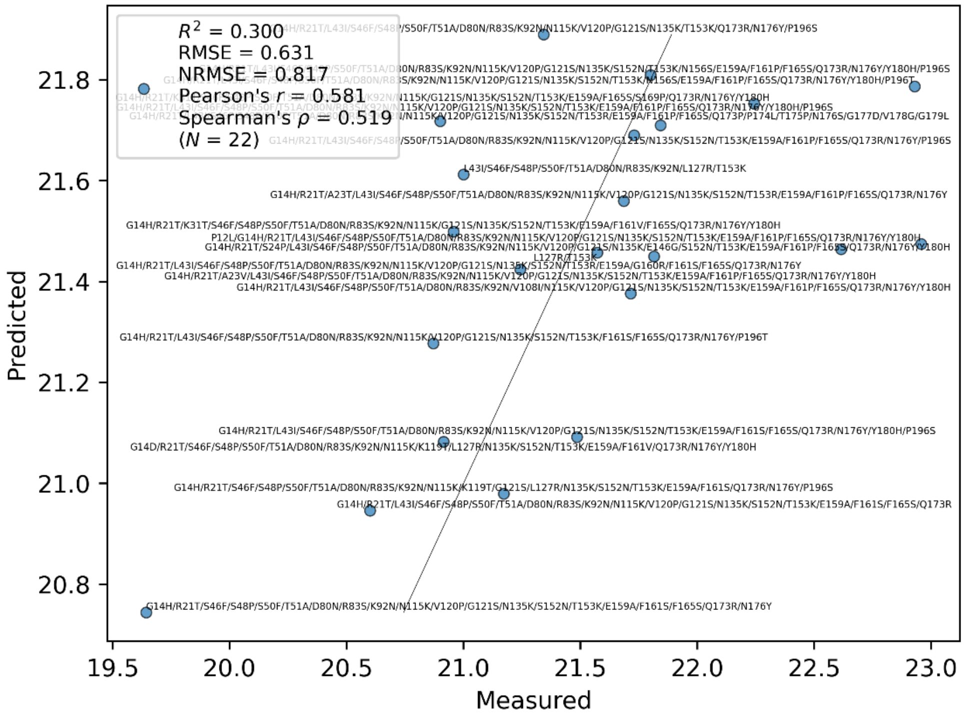

Figure S1: Regression status of each model

This set of figures is a fitting diagram generated by PyPEF. This figure fully demonstrates the difference in fitting results and the best adaptability of ISOY800101.

Restraints in ligand:			Restraints in receptor:		
1	LYS	99	1	GLN	24
2	VAL	127	2	THR	27
3	TYR	131	3	PHE	28
4	TYR	135	4	ASP	30
5	LEU	137	5	LYS	31
6	PHE	138	6	HIS	34
7	ALA	157	7	GLU	37
8	CYS	162	8	ASP	38
9	PRO	168	9	TYR	41
10	ASN	169	10	GLN	42
11	TYR	171	11	LEU	79
12	GLN	175	12	MET	82
13	GLY	178	13	ASN	330
14	GLN	180	14	LYS	353
15	THR	182	15	GLY	354
16	ASN	183	16	ARG	357
17	ASP	184	17	ARG	393
18	TYR	187			

Figure S2: Site selection for rigid docking

Table S1 Data Set： This table is the dataset used in this study.	
Variant	week/10
G14H/R21T/L43I/S46F/S48P/S50F/T51A/D80N/R83S/K92N/N115K/V120P/G121S/N135K/S152N/T153K/E159A/F161S/F165S/Q173R/N176Y/Y180H	19.35714
G14H/R21T/L43I/S46F/S48P/S50F/T51A/D80N/R83S/K92N/N115K/V120P/G121S/N135K/S152N/T153K/E159A/F161S/F165S/Q173R/N176Y	19.51429
G14H/R21T/L43I/S46F/S48P/S50F/T51A/D80N/K92N/N115K/V120P/G121S/N135K/S152N/T153K/E159A/F161S/F165S/Q173R/N176Y/Y180H	19.58571
F13X/G14H/R21T/L43I/S46F/S48P/S50F/T51A/D80N/R83S/K92N/N115K/V120P/G121S/N135K/S152N/T153K/E159A/F161S/F165S/Q173R/N176Y/Y180H	19.62857
G14H/R21T/S46F/S48P/S50F/T51A/D80N/R83S/K92N/N115K/V120P/G121S/N135K/S152N/T153K/E159A/F161S/F165S/Q173R/N176Y	19.64286
G14H/R21T/L43I/S46F/S48P/S50F/T51A/D80N/R83S/K92N/V120P/N135K/S152N/T153K/E159A/F161S/Q173R/N176Y/Y180H	20.28571
G14H/R21T/L43I/S46F/S48P/S50F/T51A/D80N/R83S/K92N/N115K/V120X/G121S/N135K/S152N/T153K/E159A/F161S/F165S/Q173R/N176Y/Y180H	20.42857
G14H/R21T/A23T/L43I/S46F/S48P/S50F/T51A/D80N/R83S/K92N/N115K/V120P/G121S/N135K/S152N/T153K/E159A/F161P/F165S/Q173R/N176Y/Y180H	20.5
G14H/R21T/L43I/S46F/S48P/S50F/T51A/D80N/R83S/K92N/N115K/V120P/G121S/N135K/S152N/T153K/E159A/F161S/F165S/Q173R	20.6
G14H/R21T/L43I/S46F/S48P/S50F/T51A/D80N/R83X/K92N/N115K/V120P/G121S/N135K/S152N/T153K/E159A/F161P/F165S/Q173R/N176Y/Y180H	20.72857
G14H/R21T/L43I/S46F/S48P/S50F/T51A/D80N/R83S/K92N/N115K/V120P/G121S/N135K/S152N/T153K/E159A/F161S/F165S/Q173X/N176Y/Y180H	20.72857
G14H/R21T/L43I/S46F/S48P/S50F/T51A/D80N/R83S/K92N/N115K/V120P/G121S/N135K/T153K/E159A/F161S/F165S/Q173R/N176Y/Y180H	20.84286
G14D/S46F/S48P/S50F/T51A/D80N/R83S/K92N/N115K/K119T/L127R/N135K/S152N/T153K/E159A/F161V/Q173R/N176Y	20.87143
G14H/R21T/L43I/S46F/S48P/S50F/T51A/D80N/R83S/K92N/N115K/V120P/G121S/N135K/S152N/T153K/F161S/F165S/Q173R/N176Y/P196T	20.87143
G14H/R21T/L43I/S46F/S48P/S50F/T51A/D80N/R83S/K92N/N115K/V120P/G121S/N135K/S152N/T153K/E159A/F161P/F165S/Q173R/N176Y	20.88571
G14H/R21T/L43I/S46F/S48P/S50F/T51A/D80N/R83S/K92N/N115K/V120P/G121S/N135K/S152N/T153K/E159A/F161P/F165S/Q173R/N176Y/Y180H	20.88571
G14H/L43I/S46F/S48P/S50F/T51A/D80N/R83S/K92N/N115K/V120P/G121S/N135K/T153K/Q173X/P174X/T175X/N176X	20.9
G14H/R21T/L43I/S46F/S48P/S50F/T51A/D80N/R83S/K92N/N115K/V120P/G121S/N135K/S152N/T153K/E159A/F161P/F165S/Q173R/N176Y/Y180H/P196S	20.9
G14H/R21T/L43I/S46F/S48P/S50F/T51A/D80N/R83S/K92N/N115K/V120P/G121S/N135K/S152N/T153K/E159A/F161S/F165S/Q173R/N176Y/P196Q	20.9
G14D/S46F/S48P/S50F/T51A/D80N/R83X/K92N/N115K/N125D/L127R/S152N/T153K/E159A/F161V/Q173R/N176Y/Y180H	20.9
G14H/R21T/S46F/S48P/S50F/T51A/D80N/R83S/K92N/N115K/K119T/G121S/L127R/N135K/S152N/T153K/E159A/F161S/Q173R/N176Y/Y180H	20.9
G14D/R21T/S46F/S48P/S50F/T51A/D80N/R83S/K92N/N115K/K119T/L127R/N135K/S152N/T153K/E159A/F161V/Q173R/N176Y/Y180H	20.91429
G14D/S46F/S48P/S50F/T51A/D80N/R83S/K92N/N115K/K119T/L127R/N135K/S152N/T153K/E159A/F161V/Q173R/N176Y/Y180H	20.91429
G14D/S46F/S48P/S50F/T51A/D80N/R83X/K92N/N115K/K119T/L127R/N135K/S152N/T153K/E159A/F161V/Q173R/N176Y/Y180H	20.91429
G14D/R21T/S46F/S48P/S50F/T51A/D80N/R83X/K92N/N115K/K119T/L127R/N135K/S152N/T153K/E159A/F161V/Q173R/N176Y/Y180H	20.92857
G14D/S46F/S48P/S50F/T51A/D80N/R83S/K92N/N115K/K119R/G121S/N125D/L127M/N135K/S152N/T153K/E159R/F161S/Q173R/N176Y/Y180H	20.94286
F13X/S46F/S48P/S50F/T51A/D80N/R83S/K92N/N115K/L127R/S152N/T153K/E159A/F161V/Q173R/N176Y/Y180H	20.94286
G14D/S46F/S48P/S50F/T51A/D80N/R83S/K92N/N115K/K119T/L127R/L130X/F131X/R132X/K133X/N135K/S152N/T153K/E159A/F161V/Q173R/N176Y/Y18[illegible]	20.94286
G14H/R21T/K31T/S46F/S48P/S50F/T51A/D80N/R83S/K92N/N115K/G121S/N135K/S152N/T153K/E159A/F161V/F165S/Q173R/N176Y/Y180H	20.95714
G14H/R21T/S46F/S48P/S50F/T51A/D80N/R83S/K92N/N115K/G121S/N135K/S152N/T153K/E159A/F161P/F165S/Q173R/N176Y/Y180H	20.95714
G14H/R21T/K31T/S46F/S48P/S50F/T51A/D80N/R83S/K92N/N115K/G121S/N135K/S152N/T153K/E159A/F165S/Q173R/N176Y/Y180H	20.98571
G14H/R21T/K31T/S46F/S48P/S50F/T51A/D80N/R83S/K92N/S113X/N115K/G121S/N135K/S152N/T153K/E159A/F165S/Q173R/N176Y/Y180H	20.98571
L43I/S46F/S48P/S50F/T51A/D80N/R83S/K92N/L127R/T153K	21
G14H/R21T/S46F/S48P/S50F/T51A/D80N/R83S/K92N/N115K/K119T/L127X/N135K/S152N/T153K/E159A/F161V/Q173R/N176Y/Y180H	21
G14D/R21T/S34N/S46F/S48P/S50F/T51A/D80N/R83X/K92N/N115K/K119T/L127R/N135K/S152N/T153K/E159A/F161V/Q173R/N176Y/Y180H	21
G14H/R21T/S46F/S48P/S50F/T51A/D80N/R83S/K92N/N115K/G121S/L127R/N135K/S152N/T153K/E159A/F161I/Q173R/N176Y/Y180H	21
G14X/R21T/L43I/S46F/S48P/S50F/T51A/D80N/R83S/K92N/N115K/V120P/G121S/N135K/S152N/T153K/E159A/F161P/F165S/Q173R/N176Y/Y180H	21
G14D/S46F/S48P/S50F/T51A/D80N/R83S/K92N/N115K/N125D/L127R/S152N/T153K/E159A/F161V/Q173R/N176Y/Y180H	21

G14H/R21T/K31T/S46F/S48P/S50F/T51A/D80N/R83S/K92N/K99X/L100X/S113X/N115K/G121S/N135K/S152N/T153K/E159A/F165S/Q173R/N176Y/Y180H	21.1
G14H/R21T/K31T/S46F/S48P/S50F/T51A/D80N/R83S/K92N/W111X/N112X/S113X/N114X/N115K/G121S/N135K/S152N/T153K/E159A/F161V/F165S/Q173R/	21.1
G14H/R21T/S46F/S48P/S50F/T51A/D80N/R83S/K92N/N115K/K119T/G121S/L127R/N135K/S152N/T153K/E159A/F161S/Q173R/N176Y/P196S	21.17143
G14H/L43I/S46F/S48P/S50F/T51A/D80N/R83S/K92N/N115K/V120P/G121S/N135K/S152N/T153K/E159A/F161P/F165S/Q173R/N176Y/Y180H	21.18571
G14D/R21T/S46F/S48P/S50F/T51A/D80N/R83S/K92N/N115K/K119T/L127R/N135K/S152N/T153K/E159A/F161V/Q173R/N176Y/Y180H/P196T	21.21429
G14H/R21T/A23V/L43I/S46F/S48P/S50F/T51A/D80N/R83S/K92N/N115K/V120P/G121S/N135K/S152N/T153K/E159A/F161P/F165S/Q173R/N176Y/Y180H	21.24286
G14H/R21T/L43I/S46F/S48P/S50F/T51A/D80N/R83S/K92N/N115K/K119T/L127R/N135K/S152N/T153K/E159A/F161V/F165S/Q173R/N176Y/Y180H	21.27143
G14H/R21T/L43I/S46F/S48P/S50F/T51A/D80N/R83S/K92N/N115K/V120P/G121S/N135K/S152N/T153K/N156T/E159A/F161P/F165S/Q173R/N176Y/Y180H	21.3
G14D/R21X/S46F/S48P/S50F/T51A/D80N/R83S/K92N/N115K/L127X/N135K/S152N/T153K/E159A/F161A/Q173R/N176Y/Y180H	21.31429
G14H/R21T/L43I/S46F/S48P/S50F/T51A/D80N/R83S/K92N/N115K/V120P/G121S/N135K/T153K/Q173R/N176Y/P196S	21.34286
G14H/R21T/L43I/S46F/S48P/S50F/T51A/D80N/R83S/K92N/N115K/V120P/G121S/N135K/S152N/T153R/E159A/F161P/F165S/Q173R/N176Y	21.37143
G14H/R21T/L43I/S46F/S48P/S50F/T51A/D80N/R83S/K92N/N115K/V120P/G121S/N135K/S152N/T153K/E159V/F161P/F165S/Q173R/N176Y/Y180H	21.38571
G14H/R21T/L43I/S46F/S48P/S50F/T51A/D80N/R83S/K92N/N115K/V120P/G121S/N135K/S152N/T153K/E159A/F161S/F165S/Q173R/N176Y/Y180H/P196S	21.48571
I1X/G14D/R21T/S46F/S48P/S50F/T51A/D80N/R83S/K92N/N115K/K119T/L127R/N135K/S152N/T153K/E159A/F161V/Q173R/N176Y/Y180H	21.48571
G14H/R21T/A23S/L43I/S46F/S48P/S50F/T51A/D80N/R83S/K92N/N115K/V120P/G121S/N135K/S152N/T153K/E159A/F161P/F165S/Q173R/N176Y/Y180H	21.5
G14H/R21T/L43I/S46F/S48P/S50F/T51A/D80N/R83S/K92N/N115K/V120X/G121S/N135K/S152N/T153K/E159A/F161P/F165S/Q173R/N176Y/Y180H	21.5
G14H/R21T/S46F/S48P/S50F/T51A/D80N/R83S/K92N/N115K/V120X/G121X/N135K/S152N/T153K/E159A/F161X/F165X/Q173R/N176Y/Y180H	21.5
L127R/S152I/T153K	21.57143
L127R/T153K	21.57143
G14H/R21T/S46F/S48P/S50F/T51A/D80N/R83N/K92N/N115K/K119T/G121S/L127R/N135K/S152N/T153K/E159A/F161S/Q173R/N176Y/Y180H	21.64286
G14H/L43I/S46F/S48P/S50F/T51A/D80N/R83S/K92N/N115K/V120P/G121S/N135K/T153K/Q173R/N176Y	21.67143
N18X/L43I/S46F/S48P/S50F/T51A/D80N/R83S/K92N/N115K/V120P/G121S/N135K/T153K/Q173R/N176Y	21.67143
G14H/R21T/A23T/L43I/S46F/S48P/S50F/T51A/D80N/R83S/K92N/N115K/V120P/G121S/N135K/S152N/T153R/E159A/F161P/F165S/Q173R/N176Y	21.68571
G14H/R21T/L43I/S46F/S48P/S50F/T51A/D80N/R83S/K92N/L127R/T153K/Q173R/N176Y/P196S	21.71429
L43I/S46F/S48P/S50F/T51A/D80N/R83S/K92N/L127R/T153K/N176Y	21.71429
G14H/R21T/L43I/S46F/S48P/S50F/T51A/D80N/R83S/K92N/V108I/N115K/V120P/G121S/N135K/S152N/T153K/E159A/F161P/F165S/Q173R/N176Y/Y180H	21.71429
G14H/F22C/L43I/S46F/S48P/S50F/T51A/D80N/R83S/K92N/N115K/V120P/G121S/N135K/T153K/Q173R/N176Y	21.72857
G14H/L43I/S46F/S48P/S50F/T51A/D80N/R83S/K92N/N115K/V120P/G121S/N135K/T153K/Q173R/N176Y/Y180H	21.72857
G14H/R21T/L43I/S46F/S48P/S50F/T51A/D80N/R83S/K92N/N115K/V120P/G121S/N135K/S152N/T153K/E159A/F161P/F165S/Q173R/N176Y/P196S	21.72857
G14H/R21T/L43I/S46F/S48P/S50F/T51A/D80N/R83S/K92N/N115K/V120P/G121S/N135K/S152N/T153R/E159A/F161P/F165S/Q173R/N176Y/Y180H	21.72857
G14H/R21T/L43I/S46F/S48P/S50F/T51A/D80N/R83S/K92N/N115K/V120P/G121S/N135K/T153K/Q173R/N176Y	21.72857
G14D/T20X/R21X/F22X/A23X/L43I/S46F/S48P/S50F/T51A/D80N/R83S/K92N/L127R/T153K/Q173R/N176Y	21.74286
G14H/R21T/L43I/S46F/S48P/S50F/T51A/D80N/R83S/K92N/N115K/V120P/G121S/N135K/T153K/Q173R/T175X/N176X/G177X/V178X/G179X/P196S	21.74286
L43I/S46F/S48P/S50F/T51A/D80N/R83S/K92N/N115K/V120P/G121S/N135K/T153K/Q173X/N176Y/Y180H	21.74286
G14H/L43I/S46F/S48P/S50F/T51A/D80N/R83S/K92N/N115K/V120P/G121S/N135K/T153K/G179X	21.78571
G14H/R21X/F22X/A23X/S24X/L43I/S46F/S48P/S50F/T51A/D80N/R83S/K92N/L127R/T153K/G171X/Q173R/P174H/N176Y	21.78571
L43I/S46F/S48P/S50F/T51A/D80N/R83S/K92N/N115K/V120P/G121S/T153K/Q173X/P174X/T175X/N176X/G177X/V178X/G179X	21.78571
G14H/R21T/L43I/S46F/S48P/S50F/T51A/D80N/R83S/K92N/N115K/V120P/G121S/N135K/S152N/T153K/N156S/E159A/F161P/F165S/Q173R/N176Y/Y180H/P1	21.8
G14H/R21T/L43I/S46F/S48P/S50F/T51A/D80N/R83S/K92N/N115K/V120P/G121S/N135K/T153K/Q173X/P174X/T175X/N176X/G177X/V178X/G179X/P196S	21.8
G14H/R21T/L43I/S46F/S48P/S50F/T51A/D80N/K92N/N115K/V120P/G121S/N135K/I147X/Y148X/Q149X/A150X/G151X/E159A/F161P/F165S/Q173R/N176Y/	21.81429

G14H/R21T/L43I/S46F/S48P/S50F/T51A/D80N/R83S/K92N/N115K/V120P/G121S/N135K/S152N/T153I/E159A/F161P/F165S/Q173R/N176Y/P196S	21.81429
G14H/R21T/L43I/S46F/S48P/S50F/T51A/D80N/R83S/K92N/N115K/V120P/G121S/N135K/S152N/T153K/E159A/F161P/F165S/S169P/Q173R/N176Y/P196S	21.81429
G14H/R21T/L43I/S46F/S48P/S50F/T51A/D80N/R83S/K92N/N115K/V120P/G121S/N135K/S152N/T153R/E159A/G160R/F161S/F165S/Q173R/N176Y	21.81429
T20X/R21X/F22X/A23X/S24X/V25X/Y26X/L43I/S46F/S48P/S50F/T51A/D80N/R83S/K92N/F104X/T105X/G106X/C107X/V108X/L127R/T153K/Q173R/N176Y	21.81429
G14H/R21T/L43I/S46F/S48P/S50F/T51A/D80N/R83S/K92N/N115K/V120P/G121S/N135K/T153K/Q173R/N176Y/Y180H/P196S	21.82857
G14H/F22C/L43I/S46F/S48P/S50F/T51A/D80N/R83S/K92N/N115K/V120P/G121S/N135K/S152N/T153R/E159A/F161P/F165S/Q173R/N176Y	21.84286
G14H/R21T/L43I/S46F/S48P/S50F/T51A/D80N/R83S/K92N/N115K/V120P/G121S/N135K/S152N/T153R/E159A/F161P/F165S/Q173P/P174L/T175P/N176S/G1	21.84286
G14H/R21T/L43I/S46F/S48P/S50F/T51A/D80N/R83S/K92N/N115K/V120P/G121S/N135K/T153K/Q173L/P174X/T175X/N176X	21.84286
G14H/R21T/L43I/S46F/S48P/S50F/T51A/Y55H/D80N/R83S/K92N/N115K/V120P/G121S/N135K/S152N/T153K/E159A/F161P/F165S/Q173R/N176Y/Y180H/P1	21.84286
G14H/R21T/L43I/S46F/S48P/S50F/T51A/D80N/R83S/K92N/N115K/V120P/G121S/N135K/S152N/T153Q/E159A/F161P/F165S/Q173R/N176Y/P196S	21.87143
G14H/R21T/L43I/S46F/S48P/S50F/T51A/D80N/R83S/K92N/N115K/V120P/G121S/N135K/S152N/T153K/N156S/E159A/F161P/F165S/Q173R/N176Y/Y180X/P1	21.9
G14H/L43I/S46F/S48P/S50F/T51A/D80N/R83S/K92N/N115K/V120P/G121S/N135K/T153K/Q173X/P174X/T175X/N176X/G177X/V178X/G179X/Y180X/Q181	21.9
G14H/R21T/K31T/S46F/S48P/S50F/T51A/D80N/R83S/K92N/N115K/G121S/N135K/S152N/T153K/E159A/F165S/S169P/Q173R/N176Y/Y180H	22.24286
I1X/G14H/R21T/L43I/S46F/S48P/S50F/T51A/D80N/R83S/K92N/N115K/V120P/G121S/N135K/S152N/T153K/E159A/F161P/F165S/Q173R/N176Y/Y180H	22.32857
P12X/G14H/V16X/R21T/L43I/S46F/S48P/S50F/T51A/D80N/R83S/K92N/N115K/V120P/G121S/N135K/S152N/T153K/E159A/F161P/F165S/Q173R/N176Y/Y18	22.38571
G14H/R21T/L43I/S46F/S48P/S50F/T51A/D80N/R83X/K92N/N115K/N135K/S152N/T153R/F165S/Q173R/N176Y/Y180H	22.44286
G14H/R21T/L43I/S46F/S48P/S50F/T51A/D80N/R83S/K92N/N115K/V120P/G121S/N135K/S152N/T153K/G157S/E159A/F161P/F165S/Q173R/N176Y/Y180H	22.45714
G14H/R21T/L43I/S46F/S48P/S50F/T51A/D80N/R83S/K92N/N115K/V120P/G121S/F131L/N135K/S152N/T153K/E159A/F161P/F165S/Q173R/N176Y/Y180H	22.48571
F4X/G14H/R21T/L43I/S46F/S48P/S50F/T51A/D80N/R83S/K92N/N115K/V120P/G121S/N135K/S152N/T153K/E159A/F161P/F165S/Q173R/N176Y/Y180H	22.58571
G14H/E15X/V16X/R21T/L43I/S46F/S48P/S50F/T51A/D80N/R83S/K92N/N115K/V120P/G121S/N135K/S152N/T153K/E159A/F161P/F165S/Q173R/N176Y/Y18	22.61429
G14H/R21T/S24P/L43I/S46F/S48P/S50F/T51A/D80N/R83S/K92N/N115K/V120P/G121S/N135K/E146G/S152N/T153K/E159A/F161P/F165S/Q173R/N176Y/Y1	22.61429
G14H/R21T/L43I/S46F/S48P/S50F/T51A/D80N/R83S/K92N/N115K/V120P/G121S/F131L/N135K/S152N/T153R/E159A/F161P/F165S/Q173R/N176Y/Y180H	22.72857
G14H/A19S/R21T/L43I/S46F/S48P/S50F/T51A/D80N/R83S/K92N/N115K/V120P/G121S/N135K/S152N/T153R/E159A/F161P/F165S/Q173R/N176Y/Y180H/P1	22.82857
G14H/R21T/L43I/S46F/S48P/S50F/T51A/D80N/R83S/K92N/N115K/V120P/G121S/N135K/S152N/T153K/N156S/E159A/F161P/F165S/Q173R/N176Y/Y180H/P	22.92857
G14H/R21T/L43I/S46F/S48P/S50F/T51A/D80N/R83S/K92N/N115K/V120P/G121S/N135K/S152N/T153K/V158L/E159A/F161P/F165S/Q173R/N176Y/Y180H	22.92857
G14H/R21T/L43I/S46F/S48P/S50F/T51A/D80N/R83S/K92N/N115K/V120P/G121S/N135K/Q149R/S152N/T153K/E159A/F161P/F165S/Q173R/N176Y/Y180H	22.94286
P12L/G14H/R21T/L43I/S46F/S48P/S50F/T51A/D80N/R83S/K92N/N115K/V120P/G121S/N135K/S152N/T153K/E159A/F161P/F165S/Q173R/N176Y/Y180H	22.95714
G14H/R21T/L43I/S46F/S48P/S50F/T51A/D80N/R83S/K92N/N115K/V120P/G121S/N135K/S152N/T153K/E159A/F161P/F165S/Q173R/N176Y/Y180X	23.37143

Table S2: High frequency mutation sites and their mutation frequencies in the dataset

Mutation	Frequency	Mutation	Frequency	Mutation	Frequency
D80N	0.98	N176Y	0.89	V120P	0.63
K92N	0.98	T153K	0.89	Y180H	0.63
S46F	0.98	Q173R	0.88	F165S	0.58
S48P	0.98	G14H	0.77	F161P	0.39
S50F	0.98	S152N	0.76	L127R	0.24
T51A	0.98	G121S	0.75	F161S	0.17
N115K	0.92	E159A	0.75	F161V	0.15
R83S	0.90	R21T	0.75	P196S	0.15
N135K	0.89	L43I	0.72	G14D	0.13

Table S3 Validation Data： This table compares the experimental and predicted timestamp data generated for validation.

ISOY800101			
Variant Number	Variant	Prediction	Experiment
1	P12L/G14H/R21T/L43I/S46F/S48P/S50F/T51A/D80N/R83S/K92N/N115K/V120P/G121S/N135K/S152N/T153K/E159A/F161P/F165S/Q173R/N176Y/Y180H	22.944748	22.95714286
2	G14H/R21T/L43I/S46F/S48P/S50F/T51A/D80N/R83S/K92N/N115K/V120P/G121S/N135K/S152N/T153R/E159A/F161P/F165S/Q173P/P174L/T175P/N176S/G177D/V	22.771482	21.84285714
3	G14H/R21T/L43I/S46F/S48P/S50F/T51A/D80N/R83S/K92N/N115K/V120P/G121S/N135K/S152N/T153K/V158L/E159A/F161P/F165S/Q173R/N176Y/Y180H	22.757896	22.92857143
4	G14H/A19S/R21T/L43I/S46F/S48P/S50F/T51A/D80N/R83S/K92N/N115K/V120P/G121S/N135K/S152N/T153R/E159A/F161P/F165S/Q173R/N176Y/Y180H/P196S	22.7291	22.82857143
5	G14H/R21T/L43I/S46F/S48P/S50F/T51A/D80N/R83S/K92N/N115K/V120P/G121S/F131L/N135K/S152N/T153R/E159A/F161P/F165S/Q173R/N176Y/Y180H	21.967904	22.72857143
6	G14H/R21T/L43I/S46F/S48P/S50F/T51A/Y55H/D80N/R83S/K92N/N115K/V120P/G121S/N135K/S152N/T153K/E159A/F161P/F165S/Q173R/N176Y/Y180H/P196S	21.943086	21.84285714
7	G14H/R21T/S24P/L43I/S46F/S48P/S50F/T51A/D80N/R83S/K92N/N115K/V120P/G121S/N135K/E146G/S152N/T153K/E159A/F161P/F165S/Q173R/N176Y/Y180H	21.903183	22.61428571
8	G14H/R21T/L43I/S46F/S48P/S50F/T51A/D80N/R83S/K92N/N115K/V120P/G121S/F131L/N135K/S152N/T153K/E159A/F161P/F165S/Q173R/N176Y/Y180H	21.84303	22.48571429
9	G14H/L43I/S46F/S48P/S50F/T51A/D80N/R83S/K92N/N115K/V120P/G121S/N135K/T153K/Q173R/N176Y/Y180H	21.828654	21.72857143
10	G14H/F22C/L43I/S46F/S48P/S50F/T51A/D80N/R83S/K92N/N115K/V120P/G121S/N135K/T153K/Q173R/N176Y	21.828342	21.72857143
11	L43I/S46F/S48P/S50F/T51A/D80N/R83S/K92N/L127R/T153K	21.798453	21
12	L43I/S46F/S48P/S50F/T51A/D80N/R83S/K92N/L127R/T153K/N176Y	21.772955	21.71428571
13	G14H/F22C/L43I/S46F/S48P/S50F/T51A/D80N/R83S/K92N/N115K/V120P/G121S/N135K/S152N/T153R/E159A/F161P/F165S/Q173R/N176Y	21.742835	21.84285714
14	G14H/R21T/L43I/S46F/S48P/S50F/T51A/D80N/R83S/K92N/N115K/V120P/G121S/N135K/S152N/T153K/E159A/F161P/F165S/Q173R/N176Y/Y180H/P196S	21.735561	20.9
15	G14H/R21T/L43I/S46F/S48P/S50F/T51A/D80N/R83S/K92N/N115K/V120P/G121S/N135K/S152N/T153R/E159A/F161P/F165S/Q173R/N176Y/Y180H	21.733938	21.72857143
16	G14H/R21T/L43I/S46F/S48P/S50F/T51A/D80N/R83S/K92N/N115K/V120P/G121S/N135K/T153K/Q173R/N176Y/Y180H/P196S	21.728617	21.82857143
17	G14H/R21T/L43I/S46F/S48P/S50F/T51A/D80N/R83S/K92N/N115K/V120P/G121S/N135K/S152N/T153K/N156S/E159A/F161P/F165S/Q173R/N176Y/Y180H/P196T	21.721655	22.92857143
18	G14H/R21T/L43I/S46F/S48P/S50F/T51A/D80N/R83S/K92N/N115K/V120P/G121S/N135K/S152N/T153K/E159A/F161P/F165S/S169P/Q173R/N176Y/P196S	21.71419	21.81428571
19	G14H/R21T/L43I/S46F/S48P/S50F/T51A/D80N/R83S/K92N/N115K/V120P/G121S/N135K/S152N/T153I/E159A/F161P/F165S/Q173R/N176Y/P196S	21.714053	21.81428571
20	G14H/R21T/L43I/S46F/S48P/S50F/T51A/D80N/R83S/K92N/N115K/V120P/G121S/N135K/S152N/T153K/N156S/E159A/F161P/F165S/Q173R/N176Y/Y180H/P196S	21.703942	21.8
21	G14H/R21T/L43I/S46F/S48P/S50F/T51A/D80N/R83S/K92N/N115K/V120P/G121S/N135K/T153K/Q173R/N176Y/P196S	21.695022	21.34285714
22	G14H/R21T/L43I/S46F/S48P/S50F/T51A/D80N/R83S/K92N/N115K/V120P/G121S/N135K/S152N/T153K/G157S/E159A/F161P/F165S/Q173R/N176Y/Y180H	21.686387	22.45714286
23	G14H/R21T/L43I/S46F/S48P/S50F/T51A/D80N/R83S/K92N/N115K/V120P/G121S/N135K/Q149R/S152N/T153K/E159A/F161P/F165S/Q173R/N176Y/Y180H	21.667944	22.94285714
24	L127R/T153K	21.655095	21.57142857
25	G14H/R21T/L43I/S46F/S48P/S50F/T51A/D80N/R83S/K92N/N115K/V120P/G121S/N135K/S152N/T153K/E159A/F161P/F165S/Q173R/N176Y/Y180H	21.635735	20.88571429
26	G14H/R21T/L43I/S46F/S48P/S50F/T51A/D80N/R83S/K92N/V108I/N115K/V120P/G121S/N135K/S152N/T153K/E159A/F161P/F165S/Q173R/N176Y/Y180H	21.63165	21.71428571
27	G14H/R21T/L43I/S46F/S48P/S50F/T51A/D80N/R83S/K92N/N115K/V120P/G121S/N135K/T153K/Q173R/N176Y	21.628143	21.72857143
28	G14H/R21T/L43I/S46F/S48P/S50F/T51A/D80N/R83S/K92N/L127R/T153K/Q173R/N176Y/P196S	21.614007	21.71428571
29	G14H/L43I/S46F/S48P/S50F/T51A/D80N/R83S/K92N/N115K/V120P/G121S/N135K/T153K/Q173R/N176Y	21.513519	21.67142857
30	G14H/R21T/L43I/S46F/S48P/S50F/T51A/D80N/R83S/K92N/N115K/V120P/G121S/N135K/S152N/T153K/E159V/F161P/F165S/Q173R/N176Y/Y180H	21.48597	21.38571429
31	G14H/R21T/L43I/S46F/S48P/S50F/T51A/D80N/R83S/K92N/N115K/V120P/G121S/N135K/S152N/T153R/E159A/F161P/F165S/Q173R/N176Y	21.471429	21.37142857
32	L127R/S152I/T153K	21.471207	21.57142857
33	G14H/R21T/L43I/S46F/S48P/S50F/T51A/D80N/R83S/K92N/N115K/V120P/G121S/N135K/S152N/T153K/E159A/F161P/F165S/Q173R/N176Y/P196S	21.460224	21.72857143

34	G14H/R21T/L43I/S46F/S48P/S50F/T51A/D80N/R83S/K92N/N115K/V120P/G121S/N135K/S152N/T153Q/E159A/F161P/F165S/Q173R/N176Y/P196S	21.447679	21.87142857
35	G14H/R21T/A23S/L43I/S46F/S48P/S50F/T51A/D80N/R83S/K92N/N115K/V120P/G121S/N135K/S152N/T153K/E159A/F161P/F165S/Q173R/N176Y/Y180H	21.400384	21.5
36	G14H/R21T/L43I/S46F/S48P/S50F/T51A/D80N/R83S/K92N/N115K/V120P/G121S/N135K/S152N/T153R/E159A/G160R/F161S/F165S/Q173R/N176Y	21.370878	21.81428571
37	G14H/R21T/L43I/S46F/S48P/S50F/T51A/D80N/R83S/K92N/N115K/V120P/G121S/N135K/S152N/T153K/E159A/F161P/F165S/Q173R/N176Y	21.36967	20.88571429
38	G14H/R21T/A23T/L43I/S46F/S48P/S50F/T51A/D80N/R83S/K92N/N115K/V120P/G121S/N135K/S152N/T153K/E159A/F161P/F165S/Q173R/N176Y/Y180H	21.321875	20.5
39	G14D/R21T/S46F/S48P/S50F/T51A/D80N/R83S/K92N/N115K/K119T/L127R/N135K/S152N/T153K/E159A/F161V/Q173R/N176Y/Y180H/P196T	21.314108	21.21428571
40	G14H/R21T/L43I/S46F/S48P/S50F/T51A/D80N/R83S/K92N/N115K/V120P/G121S/N135K/S152N/T153K/N156T/E159A/F161P/F165S/Q173R/N176Y/Y180H	21.312678	21.3
41	G14H/R21T/A23V/L43I/S46F/S48P/S50F/T51A/D80N/R83S/K92N/N115K/V120P/G121S/N135K/S152N/T153K/E159A/F161P/F165S/Q173R/N176Y/Y180H	21.309581	21.24285714
42	G14H/L43I/S46F/S48P/S50F/T51A/D80N/R83S/K92N/N115K/V120P/G121S/N135K/S152N/T153K/E159A/F161P/F165S/Q173R/N176Y/Y180H	21.285621	21.18571429
43	G14H/R21T/A23T/L43I/S46F/S48P/S50F/T51A/D80N/R83S/K92N/N115K/V120P/G121S/N135K/S152N/T153R/E159A/F161P/F165S/Q173R/N176Y	21.277498	21.68571429
44	G14H/R21T/S46F/S48P/S50F/T51A/D80N/R83N/K92N/N115K/K119T/G121S/L127R/N135K/S152N/T153K/E159A/F161S/Q173R/N176Y/Y180H	21.260644	21.64285714
45	G14H/R21T/L43I/S46F/S48P/S50F/T51A/D80N/R83S/K92N/N115K/K119T/L127R/N135K/S152N/T153K/E159A/F161V/F165S/Q173R/N176Y/Y180H	21.171035	21.27142857
46	G14H/R21T/S46F/S48P/S50F/T51A/D80N/R83S/K92N/N115K/K119T/G121S/L127R/N135K/S152N/T153K/E159A/F161S/Q173R/N176Y/Y180H	21.132411	20.9
47	G14D/R21T/S46F/S48P/S50F/T51A/D80N/R83S/K92N/N115K/K119T/L127R/N135K/S152N/T153K/E159A/F161V/Q173R/N176Y/Y180H	21.105799	20.91428571
48	G14D/S46F/S48P/S50F/T51A/D80N/R83S/K92N/N115K/N125D/L127R/S152N/T153K/E159A/F161V/Q173R/N176Y/Y180H	21.100356	21
49	G14H/R21T/S46F/S48P/S50F/T51A/D80N/R83S/K92N/N115K/G121S/L127R/N135K/S152N/T153K/E159A/F161I/Q173R/N176Y/Y180H	21.099703	21
50	G14H/R21T/S46F/S48P/S50F/T51A/D80N/R83S/K92N/N115K/K119T/G121S/L127R/N135K/S152N/T153K/E159A/F161S/Q173R/N176Y/P196S	21.0734	21.17142857
51	G14H/R21T/S46F/S48P/S50F/T51A/D80N/R83S/K92N/N115K/G121S/N135K/S152N/T153K/E159A/F161P/F165S/Q173R/N176Y/Y180H	21.057354	20.95714286
52	G14H/R21T/L43I/S46F/S48P/S50F/T51A/D80N/R83S/K92N/N115K/V120P/G121S/N135K/S152N/T153K/E159A/F161S/F165S/Q173R/N176Y/Y180H/P196S	21.033909	21.48571429
53	G14H/R21T/L43I/S46F/S48P/S50F/T51A/D80N/R83S/K92N/N115K/V120P/G121S/N135K/S152N/T153K/E159A/F161S/F165S/Q173R/N176Y/P196Q	21.000221	20.9
54	G14D/S46F/S48P/S50F/T51A/D80N/R83S/K92N/N115K/K119T/L127R/N135K/S152N/T153K/E159A/F161V/Q173R/N176Y	20.971443	20.87142857
55	G14H/R21T/L43I/S46F/S48P/S50F/T51A/D80N/R83S/K92N/N115K/V120P/G121S/N135K/T153K/E159A/F161S/F165S/Q173R/N176Y/Y180H	20.942907	20.84285714
56	G14H/R21T/L43I/S46F/S48P/S50F/T51A/D80N/R83S/K92N/N115K/V120P/G121S/N135K/S152N/T153K/F161S/F165S/Q173R/N176Y/P196T	20.942365	20.87142857
57	G14H/R21T/L43I/S46F/S48P/S50F/T51A/D80N/R83S/K92N/N115K/V120P/G121S/N135K/S152N/T153K/E159A/F161S/F165S/Q173R/N176Y/Y180H	20.936558	19.35714286
58	G14D/S46F/S48P/S50F/T51A/D80N/R83S/K92N/N115K/K119T/L127R/N135K/S152N/T153K/E159A/F161V/Q173R/N176Y/Y180H	20.925062	20.91428571
59	G14H/R21T/K31T/S46F/S48P/S50F/T51A/D80N/R83S/K92N/N115K/G121S/N135K/S152N/T153K/E159A/F165S/S169P/Q173R/N176Y/Y180H	20.923653	22.24285714
60	G14H/R21T/K31T/S46F/S48P/S50F/T51A/D80N/R83S/K92N/N115K/G121S/N135K/S152N/T153K/E159A/F165S/Q173R/N176Y/Y180H	20.885534	20.98571429
61	G14D/S46F/S48P/S50F/T51A/D80N/R83S/K92N/N115K/K119R/G121S/N125D/L127M/N135K/S152N/T153K/E159R/F161S/Q173R/N176Y/Y180H	20.84282	20.94285714
62	G14H/R21T/L43I/S46F/S48P/S50F/T51A/D80N/R83S/K92N/N115K/V120P/G121S/N135K/S152N/T153K/E159A/F161S/F165S/Q173R/N176Y	20.705304	19.51428571
63	G14H/R21T/K31T/S46F/S48P/S50F/T51A/D80N/R83S/K92N/N115K/G121S/N135K/S152N/T153K/E159A/F161V/F165S/Q173R/N176Y/Y180H	20.667012	20.95714286
64	G14H/R21T/L43I/S46F/S48P/S50F/T51A/D80N/R83S/K92N/N115K/V120P/G121S/N135K/S152N/T153K/E159A/F161S/F165S/Q173R	20.666287	20.6
65	G14H/R21T/L43I/S46F/S48P/S50F/T51A/D80N/R83S/K92N/V120P/N135K/S152N/T153K/E159A/F161S/Q173R/N176Y/Y180H	20.385832	20.28571429
66	G14H/R21T/S46F/S48P/S50F/T51A/D80N/R83S/K92N/N115K/V120P/G121S/N135K/S152N/T153K/E159A/F161S/F165S/Q173R/N176Y	20.247648	19.64285714
67	G14H/R21T/L43I/S46F/S48P/S50F/T51A/D80N/K92N/N115K/V120P/G121S/N135K/S152N/T153K/E159A/F161S/F165S/Q173R/N176Y/Y180H	19.685956	19.58571429

QIAN880132			
Variant Number	Variant	Prediction	Experiment
1	G14H/A19S/R21T/L43I/S46F/S48P/S50F/T51A/D80N/R83S/K92N/N115K/V120P/G121S/N135K/S152N/T153R/E159A/F161P/F165S/Q173R/N176Y/Y180H/P196S	22.728776	22.82857143
2	G14H/R21T/L43I/S46F/S48P/S50F/T51A/D80N/R83S/K92N/N115K/V120P/G121S/N135K/S152N/T153R/E159A/F161P/F165S/Q173P/P174L/T175P/N176S/G177[illegible]	22.301789	21.84285714
3	G14H/R21T/L43I/S46F/S48P/S50F/T51A/D80N/R83S/K92N/N115K/V120P/G121S/N135K/S152N/T153K/G157S/E159A/F161P/F165S/Q173R/N176Y/Y180H	22.220591	22.45714286
4	G14H/R21T/L43I/S46F/S48P/S50F/T51A/D80N/R83S/K92N/N115K/V120P/G121S/N135K/S152N/T153I/E159A/F161P/F165S/Q173R/N176Y/P196S	22.106037	21.81428571
5	P12L/G14H/R21T/L43I/S46F/S48P/S50F/T51A/D80N/R83S/K92N/N115K/V120P/G121S/N135K/S152N/T153K/E159A/F161P/F165S/Q173R/N176Y/Y180H	22.01303	22.95714286
6	G14H/R21T/L43I/S46F/S48P/S50F/T51A/D80N/R83S/K92N/N115K/V120P/G121S/N135K/S152N/T153K/N156S/E159A/F161P/F165S/Q173R/N176Y/Y180H/P19[illegible]	21.881469	22.92857143
7	G14H/R21T/L43I/S46F/S48P/S50F/T51A/D80N/R83S/K92N/N115K/V120P/G121S/N135K/S152N/T153K/E159A/F161P/F165S/S169P/Q173R/N176Y/P196S	21.823371	21.81428571
8	G14H/R21T/L43I/S46F/S48P/S50F/T51A/Y55H/D80N/R83S/K92N/N115K/V120P/G121S/N135K/S152N/T153K/E159A/F161P/F165S/Q173R/N176Y/Y180H/P196[illegible]	21.811722	21.84285714
9	G14H/R21T/L43I/S46F/S48P/S50F/T51A/D80N/R83S/K92N/N115K/V120P/G121S/N135K/S152N/T153Q/E159A/F161P/F165S/Q173R/N176Y/P196S	21.771058	21.87142857
10	G14H/F22C/L43I/S46F/S48P/S50F/T51A/D80N/R83S/K92N/N115K/V120P/G121S/N135K/S152N/T153R/E159A/F161P/F165S/Q173R/N176Y	21.742785	21.84285714
11	G14H/R21T/L43I/S46F/S48P/S50F/T51A/D80N/R83S/K92N/N115K/V120P/G121S/N135K/T153K/Q173R/N176Y/Y180H/P196S	21.728759	21.82857143
12	G14H/L43I/S46F/S48P/S50F/T51A/D80N/R83S/K92N/N115K/V120P/G121S/N135K/T153K/Q173R/N176Y/Y180H	21.702097	21.72857143
13	G14H/R21T/L43I/S46F/S48P/S50F/T51A/D80N/R83S/K92N/N115K/V120P/G121S/N135K/T153K/Q173R/N176Y	21.682367	21.72857143
14	L127R/S152I/T153K	21.671877	21.57142857
15	G14H/R21T/L43I/S46F/S48P/S50F/T51A/D80N/R83S/K92N/N115K/V120P/G121S/F131L/N135K/S152N/T153R/E159A/F161P/F165S/Q173R/N176Y/Y180H	21.664971	22.72857143
16	G14H/R21T/S24P/L43I/S46F/S48P/S50F/T51A/D80N/R83S/K92N/N115K/V120P/G121S/N135K/E146G/S152N/T153K/E159A/F161P/F165S/Q173R/N176Y/Y180H	21.64778	22.61428571
17	G14H/R21T/L43I/S46F/S48P/S50F/T51A/D80N/R83S/K92N/N115K/V120P/G121S/N135K/S152N/T153K/E159A/F161P/F165S/Q173R/N176Y/Y180H/P196S	21.647047	20.9
18	G14H/F22C/L43I/S46F/S48P/S50F/T51A/D80N/R83S/K92N/N115K/V120P/G121S/N135K/T153K/Q173R/N176Y	21.628782	21.72857143
19	G14H/R21T/L43I/S46F/S48P/S50F/T51A/D80N/R83S/K92N/N115K/V120P/G121S/N135K/S152N/T153R/E159A/F161P/F165S/Q173R/N176Y/Y180H	21.628618	21.72857143
20	G14H/R21T/L43I/S46F/S48P/S50F/T51A/D80N/R83S/K92N/L127R/T153K/Q173R/N176Y/P196S	21.614859	21.71428571
21	L43I/S46F/S48P/S50F/T51A/D80N/R83S/K92N/L127R/T153K/N176Y	21.614059	21.71428571
22	G14H/R21T/L43I/S46F/S48P/S50F/T51A/D80N/R83S/K92N/N115K/V120P/G121S/N135K/S152N/T153K/N156S/E159A/F161P/F165S/Q173R/N176Y/Y180H/P19[illegible]	21.611968	21.8
23	G14H/L43I/S46F/S48P/S50F/T51A/D80N/R83S/K92N/N115K/V120P/G121S/N135K/T153K/Q173R/N176Y	21.571834	21.67142857
24	G14H/R21T/A23T/L43I/S46F/S48P/S50F/T51A/D80N/R83S/K92N/N115K/V120P/G121S/N135K/S152N/T153R/E159A/F161P/F165S/Q173R/N176Y	21.564687	21.68571429
25	G14H/R21T/L43I/S46F/S48P/S50F/T51A/D80N/R83S/K92N/N115K/V120P/G121S/N135K/Q149R/S152N/T153K/E159A/F161P/F165S/Q173R/N176Y/Y180H	21.554642	22.94285714
26	G14H/R21T/L43I/S46F/S48P/S50F/T51A/D80N/R83S/K92N/N115K/V120P/G121S/N135K/S152N/T153K/E159A/F161P/F165S/Q173R/N176Y/P196S	21.523667	21.72857143
27	G14H/R21T/L43I/S46F/S48P/S50F/T51A/D80N/R83S/K92N/N115K/V120P/G121S/N135K/S152N/T153R/E159A/F161P/F165S/Q173R/N176Y	21.512653	21.37142857
28	G14H/R21T/L43I/S46F/S48P/S50F/T51A/D80N/R83S/K92N/N115K/V120P/G121S/N135K/T153K/Q173R/N176Y/P196S	21.488359	21.34285714
29	L43I/S46F/S48P/S50F/T51A/D80N/R83S/K92N/L127R/T153K	21.418949	21
30	G14H/R21T/A23S/L43I/S46F/S48P/S50F/T51A/D80N/R83S/K92N/N115K/V120P/G121S/N135K/S152N/T153K/E159A/F161P/F165S/Q173R/N176Y/Y180H	21.399839	21.5
31	G14H/R21T/S46F/S48P/S50F/T51A/D80N/R83N/K92N/N115K/K119T/G121S/L127R/N135K/S152N/T153K/E159A/F161S/Q173R/N176Y/Y180H	21.386046	21.64285714
32	G14D/R21T/S46F/S48P/S50F/T51A/D80N/R83S/K92N/N115K/K119T/L127R/N135K/S152N/T153K/E159A/F161V/Q173R/N176Y/Y180H/P196T	21.314379	21.21428571
33	G14H/R21T/L43I/S46F/S48P/S50F/T51A/D80N/R83S/K92N/N115K/V120P/G121S/N135K/S152N/T153K/E159V/F161P/F165S/Q173R/N176Y/Y180H	21.28557	21.38571429
34	G14H/L43I/S46F/S48P/S50F/T51A/D80N/R83S/K92N/N115K/V120P/G121S/N135K/S152N/T153K/E159A/F161P/F165S/Q173R/N176Y/Y180H	21.285515	21.18571429
35	G14H/R21T/L43I/S46F/S48P/S50F/T51A/D80N/R83S/K92N/N115K/V120P/G121S/N135K/S152N/T153K/V158L/E159A/F161P/F165S/Q173R/N176Y/Y180H	21.284091	22.92857143
36	G14H/R21T/A23T/L43I/S46F/S48P/S50F/T51A/D80N/R83S/K92N/N115K/V120P/G121S/N135K/S152N/T153K/E159A/F161P/F165S/Q173R/N176Y/Y180H	21.236337	20.5

37	G14H/R21T/L43I/S46F/S48P/S50F/T51A/D80N/R83S/K92N/N115K/V120P/G121S/F131L/N135K/S152N/T153K/E159A/F161P/F165S/Q173R/N176Y/Y180H	21.22645	22.48571429
38	G14H/R21T/L43I/S46F/S48P/S50F/T51A/D80N/R83S/K92N/N115K/V120P/G121S/N135K/S152N/T153K/N156T/E159A/F161P/F165S/Q173R/N176Y/Y180H	21.19994	21.3
39	G14H/R21T/L43I/S46F/S48P/S50F/T51A/D80N/R83S/K92N/N115K/V120P/G121S/N135K/S152N/T153K/E159A/F161P/F165S/Q173R/N176Y/Y180H	21.190593	20.88571429
40	G14H/R21T/L43I/S46F/S48P/S50F/T51A/D80N/R83S/K92N/N115K/K119T/L127R/N135K/S152N/T153K/E159A/F161V/F165S/Q173R/N176Y/Y180H	21.171184	21.27142857
41	G14H/R21T/L43I/S46F/S48P/S50F/T51A/D80N/R83S/K92N/V108I/N115K/V120P/G121S/N135K/S152N/T153K/E159A/F161P/F165S/Q173R/N176Y/Y180H	21.152518	21.71428571
42	G14H/R21T/K31T/S46F/S48P/S50F/T51A/D80N/R83S/K92N/N115K/G121S/N135K/S152N/T153K/E159A/F165S/S169P/Q173R/N176Y/Y180H	21.141607	22.24285714
43	G14D/S46F/S48P/S50F/T51A/D80N/R83S/K92N/N115K/N125D/L127R/S152N/T153K/E159A/F161V/Q173R/N176Y/Y180H	21.099786	21
44	G14H/R21T/A23V/L43I/S46F/S48P/S50F/T51A/D80N/R83S/K92N/N115K/V120P/G121S/N135K/S152N/T153K/E159A/F161P/F165S/Q173R/N176Y/Y180H	21.089292	21.24285714
45	G14H/R21T/L43I/S46F/S48P/S50F/T51A/D80N/R83S/K92N/N115K/V120P/G121S/N135K/S152N/T153K/E159A/F161P/F165S/Q173R/N176Y	21.066766	20.88571429
46	G14H/R21T/S46F/S48P/S50F/T51A/D80N/R83S/K92N/N115K/G121S/N135K/S152N/T153K/E159A/F161P/F165S/Q173R/N176Y/Y180H	21.057234	20.95714286
47	G14D/S46F/S48P/S50F/T51A/D80N/R83S/K92N/N115K/K119R/G121S/N125D/L127M/N135K/S152N/T153K/E159R/F161S/Q173R/N176Y/Y180H	21.042702	20.94285714
48	G14H/R21T/S46F/S48P/S50F/T51A/D80N/R83S/K92N/N115K/G121S/L127R/N135K/S152N/T153K/E159A/F161I/Q173R/N176Y/Y180H	21.031587	21
49	G14H/R21T/L43I/S46F/S48P/S50F/T51A/D80N/R83S/K92N/N115K/V120P/G121S/N135K/S152N/T153K/F161S/F165S/Q173R/N176Y/P196T	21.021116	20.87142857
50	G14D/R21T/S46F/S48P/S50F/T51A/D80N/R83S/K92N/N115K/K119T/L127R/N135K/S152N/T153K/E159A/F161V/Q173R/N176Y/Y180H	21.018019	20.91428571
51	G14D/S46F/S48P/S50F/T51A/D80N/R83S/K92N/N115K/K119T/L127R/N135K/S152N/T153K/E159A/F161V/Q173R/N176Y/Y180H	21.01453	20.91428571
52	G14H/R21T/L43I/S46F/S48P/S50F/T51A/D80N/R83S/K92N/N115K/V120P/G121S/N135K/S152N/T153K/E159A/F161S/F165S/Q173R/N176Y/P196Q	21.000155	20.9
53	G14H/R21T/S46F/S48P/S50F/T51A/D80N/R83S/K92N/N115K/K119T/G121S/L127R/N135K/S152N/T153K/E159A/F161S/Q173R/N176Y/Y180H	20.999668	20.9
54	G14H/R21T/K31T/S46F/S48P/S50F/T51A/D80N/R83S/K92N/N115K/G121S/N135K/S152N/T153K/E159A/F161V/F165S/Q173R/N176Y/Y180H	20.977562	20.95714286
55	L127R/T153K	20.945878	21.57142857
56	G14D/S46F/S48P/S50F/T51A/D80N/R83S/K92N/N115K/K119T/L127R/N135K/S152N/T153K/E159A/F161V/Q173R/N176Y	20.905308	20.87142857
57	G14H/R21T/S46F/S48P/S50F/T51A/D80N/R83S/K92N/N115K/K119T/G121S/L127R/N135K/S152N/T153K/E159A/F161S/Q173R/N176Y/P196S	20.903401	21.17142857
58	G14H/R21T/K31T/S46F/S48P/S50F/T51A/D80N/R83S/K92N/N115K/G121S/N135K/S152N/T153K/E159A/F165S/Q173R/N176Y/Y180H	20.885432	20.98571429
59	G14H/R21T/L43I/S46F/S48P/S50F/T51A/D80N/R83S/K92N/N115K/V120P/G121S/N135K/S152N/T153K/E159A/F161S/F165S/Q173R/N176Y/Y180H/P196S	20.876965	21.48571429
60	G14H/R21T/L43I/S46F/S48P/S50F/T51A/D80N/R83S/K92N/N115K/V120P/G121S/N135K/S152N/T153R/E159A/G160R/F161S/F165S/Q173R/N176Y	20.82189	21.81428571
61	G14H/R21T/L43I/S46F/S48P/S50F/T51A/D80N/R83S/K92N/N115K/V120P/G121S/N135K/T153K/E159A/F161S/F165S/Q173R/N176Y/Y180H	20.780254	20.84285714
62	G14H/R21T/L43I/S46F/S48P/S50F/T51A/D80N/R83S/K92N/N115K/V120P/G121S/N135K/S152N/T153K/E159A/F161S/F165S/Q173R/N176Y/Y180H	20.495018	19.35714286
63	G14H/R21T/L43I/S46F/S48P/S50F/T51A/D80N/R83S/K92N/V120P/N135K/S152N/T153K/E159A/F161S/Q173R/N176Y/Y180H	20.385706	20.28571429
64	G14H/R21T/L43I/S46F/S48P/S50F/T51A/D80N/R83S/K92N/N115K/V120P/G121S/N135K/S152N/T153K/E159A/F161S/F165S/Q173R/N176Y	20.36888	19.51428571
65	G14H/R21T/S46F/S48P/S50F/T51A/D80N/R83S/K92N/N115K/V120P/G121S/N135K/S152N/T153K/E159A/F161S/F165S/Q173R/N176Y	20.239549	19.64285714
66	G14H/R21T/L43I/S46F/S48P/S50F/T51A/D80N/R83S/K92N/N115K/V120P/G121S/N135K/S152N/T153K/E159A/F161S/F165S/Q173R	20.126684	20.6
67	G14H/R21T/L43I/S46F/S48P/S50F/T51A/D80N/K92N/N115K/V120P/G121S/N135K/S152N/T153K/E159A/F161S/F165S/Q173R/N176Y/Y180H	19.68554	19.58571429
